# Supplementary material for: Swine growth promotion with antibiotics or alternatives can increase antibiotic resistance gene mobility potential
Source: Sci Rep. 2021 Mar 9;11:5485. doi: 10.1038/s41598-021-84759-9 (PMC7970892; doi:10.1038/s41598-021-84759-9)
Supplement: Supplementary file 1 — Supplementary information. [file 41598_2021_84759_MOESM1_ESM.pdf]

Supplementary material for:

Swine Growth Promotion with Antibiotics or Alternatives can Increase Antibiotic Resistance Gene Mobility Potential

Johanna Muurinen,[a]* Jacob Richert,[b] Carmen Wickware,[a] Brian Richert, [a] and Timothy A. Johnson[a]*

[a]Department of Animal Sciences, Purdue University, West Lafayette, Indiana, USA

[b]Department of Animal Sciences & Industry, Kansas State University, Manhattan, Kansas, USA

Running Title: Growth Promoters Mobilize Antimicrobial Resistance

*Address correspondence to Johanna Muurinen and Timothy Johnson,

johanna.muurinen@onehealth.fi and john2185@purdue.edu, respectively.

Table S1. List of the used primer sets.

Assay name	Mechanism	Classification	Forward Primer	Reverse Primer
16S old 1_1	none	housekeeping	GGGTTGCGCTCGTTGC	ATGGYTGTCGTCAGCTCGTG
16S new 2_2	none	housekeeping	CCTACGGGAGGCAGCAG	ATTACCGCGGCTGCTGGC
aacC2_3	deactivate	Aminoglycoside	ACGGCATTCTCGATTGCTTT	CCGAGCTTCACGTAAGCATTT
aacA/aphD_6	deactivate	Aminoglycoside	AGAGCCTTGGGAAGATGAAGTTT	TTGATCCATACCATAGACTATCTCATCA
aac(6)-II_8	deactivate	Aminoglycoside	CGACCCGACTCCGAACAA	GCACGAATCCTGCCTTCTCA
acrB_9	efflux	MDR	AGTCGGTGTTCGCCGTTAAC	CAAGGAAACGAACGCAATACC
acrF_11	efflux	MDR	GCGGCCAGGCACAAAA	TACGCTCTTCCCACGGTTTC
adeA_12	efflux	MDR	CAGTTCGAGCGCCTATTTCTG	CGCCCTGACCGACCAAT
aphA3_14	deactivate	Aminoglycoside	AAAAGCCCGAAGAGGAACTTG	CATCTTTCACAAAGATGTTGCTGTCT
ermK_17	protection	MLSB	GTTTGATATTGGCATTGTCAGAGAAA	ACCATTGCCGAGTCCACTTT
multidrug resistance _20	efflux	MDR	AATTTTGCCGATTATTGCTGAAA	GATTGTCATCATTCGTTTATCACCAA
tet(36)_22	protection	Tetracycline	AGAATACTCAGCAGAGGTCAGTTCCT	TGGTAGGTCGATAACCCGAAAAT
erm(F)_23	protection	MLSB	CAGCTTTGGTTGAACATTTACGAA	AAATTCCTAAAATCACAACCGACAA
cfiA_24	deactivate	Beta-lactam	GCAGCGTTGCTGGACACA	GTTCGGGATAAACGTGGTGACT
Tp614_25	MGE	Transposase	GGAAATCAACGGCATCCAGTT	CATCCATGCGCTTTTGTCTCT
IS613_26	MGE	Transposase	AGGTTCGGACTCAATGCAACA	TTCAGCACATACCGCCTTGAT
blaACC-1_28	deactivate	Beta-lactam	CACACAGCTGATGGCTTATCTAAAA	AATAAACGCGATGGGTTCCA
blaMOX/blaCMY_34	deactivate	Beta-lactam	CTATGTCAATGTGCCGAAGCA	GGCTTGTCCTCTTTCGAATAGC
blaOCH_35	deactivate	Beta-lactam	GGCGACTTGCGCCGTAT	TTTTCTGCTCGGCCATGAG
blaPAO/PDC_36	deactivate	Beta-lactam	CGCCGTACAACCGGTGAT	GAAGTAATGCGGTTCTCCTTTCA
blaVEB_38	deactivate	Beta-lactam	CCCGATGCAAAGCGTTATG	GAAAGATTCCCTTTATCTATCTCAGACAA
bla1_39	deactivate	Beta-lactam	GCAAGTTGAAGCGAAAGAAAAGA	TACCAGTATCAATCGCATATACACCTAA
blaROB_41	deactivate	Beta-lactam	GCAAAGGCATGACGATTGC	CGCGCTGTTGTCGCTAAA
blaOXY-2_42	deactivate	Beta-lactam	CGTTCAGGCGGCAGGTT	GCCGCGATATAAGATTTGAGAATT
blaPSE_43	deactivate	Beta-lactam	TTGTGACCTATTCCCCTGTAATAGAA	TGCGAAGCACGCATCATC
cphA_46	deactivate	Beta-lactam	GCGAGCTGCACAAGCTGAT	CGGCCCAGTCGCTCTTC
bla-L1_48	deactivate	Beta-lactam	CACCGGGTTACCAGCTGAAG	GCGAAGCTGCGCTTGTAGTC
sat4_49	deactivate	Aminoglycoside	GAATGGGCAAAGCATAAAAACTTG	CCGATTTTGAAACCACAATTATGATA
catB3_51	catB3	Amphenicol	GCACTCGATGCCTTCCAAAA	AGAGCCGATCCAAACGTCAT
catB8_52	catB8	Amphenicol	CACTCGACGCCTTCCAAAG	CCGAGCCTATCCAGACATCATT
ceoA_53	efflux	Amphenicol	ATCAACACGGACCAGGACAAG	GGAAAGTCCGCTCACGATGA
tet(32)_54	protection	Tetracycline	CCATTACTTCGGACAACGGTAGA	CAATCTCTGTGAGGGCATTTAACA
cmr_57	efflux	MDR	CGGCATCGTCAGTGGAATT	CGGTTCCGAAAAAGATGGAA
dfrA1_58	protection	Trimethoprim	GGAATGGCCCTGATATTCCA	AGTCTTGCGTCCAACCAACAG
dfrA12_59	protection	Trimethoprim	CCTCTACCGAACCGTCACACA	GCGACAGCGTTGAAACAACTAC
acrA_62	efflux	MDR	GGTCTATCACCCTACGCGCTATC	GCGCGCACGAACATACC
emrD_64	efflux	MDR	CTCAGCAGTATGGTGGTAAGCATT	ACCAGGCGCCGAAGAAC
tetU_69	unknown	Tetracycline	GTGGCAAAGCAACGGATTG	TGCGGGCTTGCAAAACTATC
vanC_71	protection	Vancomycin	CCTGCCACAATCGATCGTT	CGGCTTCATTCGGCTTGATA
lmrA_77	efflux	MLSB	TTCAGATGCAATGGCGTTTG	ATAATCGGGAACATAATGAGCATAACTAC
nisB_79	deactivate	other	GGGAGAGTTGCCGATGTTGTA	AGCCACTCGTTAAAGGGCAAT
mdtE/yhiU_81	efflux	MDR	CGTCGGCGCACTCGTT	TCCAGACGTTGTACGGTAACCA
mexA_89	efflux	MDR	AGGACAACGCTATGCAACGAA	CCGGAAAGGGCCGAAAT

erm(36)_91	protection	MLSB	GGCGGACCGACTTGCAT	TCTGCGTTGACGACGGTTAC
aac(6)-Ib_95	deactivate	Aminoglycoside	CGTCGCCGAGCAACTTG	CGGTACCTTGCCTCTCAAACC
aadA2_97	deactivate	Aminoglycoside	ACGGCTCCGCAGTGGAT	GGCCACAGTAACCAACAAATCA
aadA5_98	deactivate	Aminoglycoside	ATCACGATCTTGCGATTTTGCT	CTGCGGATGGGCCTAGAAG
aadA_99_99	deactivate	Aminoglycoside	GTTGTGCACGACGACATCATT	GGCTCGAAGATACCTGCAAGAA
acrR_102	regulator	MDR	GCGCTGGAGACACGACAAC	GCCTTGCTGCGAGAACAAA
aph(2)-Id_104	deactivate	Aminoglycoside	TGAGCAGTATCATAAGTTGAGTGAAAAG	GACAGAACAATCAATCTCTATGGAATG
cfxA_106	deactivate	Beta-lactam	TCATTCCTCGTTCAAGTTTTCAGA	TGCAGCACCAAGAGGAGATGT
cepA_107	deactivate	Beta-lactam	AGTTGCGCAGAACAGTCCTCTT	TCGTATCTTGCCCGTCGATAAT
blaCMY_108	deactivate	Beta-lactam	AAAGCCTCATGGGTGCATAAA	ATAGCTTTTGTTTGCCAGCATCA
ampC/blaDHA_112	deactivate	Beta-lactam	TGGCCGCAGCAGAAAGA	CCGTTTTATGCACCCAGGAA
blaGES_120	deactivate	Beta-lactam	GCAATGTGCTCAACGTTCAAG	GTGCCTGAGTCAATTCTTTCAAAG
blaSFO_121	deactivate	Beta-lactam	CCGCCGCCATCCAGTA	GGGCCGCCAAGATGCT
blaTLA_122	deactivate	Beta-lactam	ACACTTTGCCATTGCTGTTTATGT	TGCAAATTTCGGCAATAATCTTT
blaZ_123	deactivate	Beta-lactam	GGAGATAAAGTAACAAATCCAGTTAGATATG	TGCTTAATTTTCCATTTGCGATAAG
qacF/H_126	efflux	MDR	TCGCAACATCCGCATTAAAA	ATGGATTTCAGAACCAGAGAAAGAAA
cmlA1_127	efflux	Amphenicol	TAGGAAGCATCGGAACGTTGAT	CAGACCGAGCACGACTGTTG
cmx(A)_129	efflux	Amphenicol	GCGATCGCCATCCTCTGT	TCGACACGGAGCCTTGGT
catA1_130	deactivate	Amphenicol	GGGTGAGTTTCACCAGTTTTGATT	CACCTTGTCGCCTTGCGTATA
sul2_133	protection	Sulfonamide	TCATCTGCCAAACTCGTCGTTA	GTCAAAGAACGCCGCAATGT
ermT_137	protection	MLSB	GTTCACTAGCACTATTTTTAATGACAGAAGT	GAAGGGTGTCTTTTTAATACAATTAACGA
msr(C)_138	protection	MLSB	TCAGACCGGATCGGTTGTC	CCTATTTTTTGGAGTCTTCTCTCTAATGTT
mphB_141	deactivate	MLSB	CGCAGCGCTTGATCTTGTAG	TTACTGCATCCATACGCTGCTT
blaVIM_147	deactivate	Beta-lactam	GCACTTCTCGCGGAGATTG	CGACGGTGATGCGTACGTT
msr(A)_149	efflux	MLSB	CTGCTAACACAAGTACGATTCCAAAT	TCAAGTAAAGTTGTCTTACCTACACCATT
aadD_151	deactivate	Aminoglycoside	CCGACAACATTTCTACCATCCTT	ACCGAAGCGCTCGTCGTATA
nimE_152	deactivate	MDR	TGCGCCAAGATAGGGCATA	GTCGTGAATTCGGCAGGTTTA
Pbp5_153	protection	Beta-lactam	GGCGAACTTCTAATTAATCCTATCCA	CGCCGATGACATTCTTCTTATCTT
pbp_154	protection	Beta-lactam	CCGGTGCCATTGGTTTAGA	AAAATAGCCGCCCCAAGATT
mecA_155	protection	Beta-lactam	GGTTACGGACAAGGTGAAATACTGAT	TGTCTTTTAATAAGTGAGGTGCGTTAATA
emrB/qacA_156	efflux	MDR	CTTTTCTCTAACCGTACATTATCTACGATAA	AGAACGTAGCGACTGATAAAATGCT
blaCTX-M_162	deactivate	Beta-lactam	GCGATAACGTGGCGATGAAT	GTCGAGACGGAACGTTTCGT
aadA2_167	deactivate	Aminoglycoside	CAATGACATTCTTGCGGGTATC	GACCTACCAAGGCAACGCTATG
aadA9_168	deactivate	Aminoglycoside	CGCGGCAAGCCTATCTTG	CAAATCAGCGACCGCAGACT
aphA1_170	deactivate	Aminoglycoside	TGAACAAGTCTGGAAAGAAATGCA	CCTATTAATTTCCCCTCGTCAAAAA
aadE_174	deactivate	Aminoglycoside	TACCTTATTGCCCTTGGAAGAGTTA	GGAACTATGTCCCTTTTAATTCTACAATCT
str_175	deactivate	Aminoglycoside	AATGAGTTTTGGAGTGTCTCAACGTA	AATCAAAACCCCTATTAAAGCCAAT
strA_176	deactivate	Aminoglycoside	CCGGTGGCATTTGAGAAAAA	GTGGCTCAACCTGCGAAAAG
strB_177	protection	Sulfonamide	GCTCGGTCGTGAGAACAATCT	CAATTTCGGTCGCCTGGTAGT
tetA_180	efflux	Tetracycline	CTCACCAGCCTGACCTCGAT	CACGTTGTTATAGAAGCCGCATAG
tetB_181	efflux	Tetracycline	AGTGCGCTTTGGATGCTGTA	AGCCCCAGTAGCTCCTGTGA
tetK_184	efflux	Tetracycline	CAGCAGTCATTGGAAAATTATCTGATTATA	CCTTGTACTAACCTACCAAAAATCAAAATA
tetQ_185	protection	Tetracycline	CGCCTCAGAAGTAAGTTCATACACTAAG	TCGTTCATGCGGATATTATCAGAAT
tetH_187	efflux	Tetracycline	TTTGGGTCATCTTACCAGCATTAA	TTGCGCATTATCATCGACAGA
tetW_191	protection	Tetracycline	ATGAACATTCCCACCGTTATCTTT	ATATCGGCGGAGAGCTTATCC

tetO_192	protection	Tetracycline	CAACATTAACGGAAAGTTTATTGTATACCA	TTGACGCTCCAAATTCATTGTATC
tetL_195	efflux	Tetracycline	ATGGTTGTAGTTGCGCGCTATAT	ATCGCTGGACCGACTCCTT
tetX_196	deactivate	Tetracycline	AAATTTGTTACCGACACGGAAGTT	CATAGCTGAAAAAATCCAGGACAGTT
tetC_199	efflux	Tetracycline	ACTGGTAAGGTAAACGCCATTGTC	ATGCATAAACCAGCCATTGAGTAAG
tetS_200	protection	Tetracycline	TTAAGGACAAACTTTCTGACGACATC	TGTCTCCCATTGTTCTGGTTCA
tnpA_201	MGE	Transposase	GCCGCACTGTCGATTTTTATC	GCGGGATCTGCCACTTCTT
tnpA_202	MGE	Transposase	CCGATCACGGAAAGCTCAAG	GGCTCGCATGACTTCGAATC
tnpA_203	MGE	Transposase	GGGCGGGTCGATTGAAA	GTGGGCGGGATCTGCTT
tnpA_204	MGE	Transposase	CATCATCGGACGGACAGAATT	GTCGGAGATGTGGGTGTAGAAAGT
tnpA_205	MGE	Transposase	GAAACCGATGCTACAATATCCAATTT	CAGCACCGTTTGCAGTGTAAG
tnpA_206	MGE	Transposase	TGCAGATGGTTTAACCTTGGATATTT	TCGGTTCATCAAACTGCTTCAC
tnpA_207	MGE	Transposase	AATTGATGCGGACGGCTTAA	TCACCAAACTGTTTATGGAGTCGTT
folA_208	protection	Sulfonamide	CGAGCAGTTCCTGCCAAAG	CCCAGTCATCCGGTTCATAATC
ermX_209	protection	MLSB	GCTCAGTGGTCCCCATGGT	ATCCCCCCGTCAACGTTT
VanB_211	protection	Vancomycin	TTGTCGGCGAAGTGGATCA	AGCCTTTTTCCGGCTCGTT
vanD_213	protection	Vancomycin	CAGAGGAACATAATGTTTCGATAAAATCT	GCCGGATTTTGTGATTCCAA
vanHD_214	protection	Vancomycin	GTGGCCGATTATACCGTCATG	CGCAGGTCATTCAGGCAAT
vanHB_215	protection	Vancomycin	GAGGTTTCCGAGGCGACAA	CTCTCGGCGGCAGTCGTAT
vanRA_216	protection	Vancomycin	CCCTTACTCCCACCGAGTTTT	TTCGTCGCCCCATATCTCAT
vanSA_218	protection	Vancomycin	CGCGTCATGCTTTCAAAATTC	TCCGCAGAAAGCTCAATTTGTT
vanWB_220	protection	Vancomycin	CGGACAAAGATACCCCCTATAAAG	AAATAGTAAATTGCTCATCTGGCACAT
vanXB_223	protection	Vancomycin	AGGCACAAAATCGAAGATGCTT	GGGTATGGCTCATCAATCAACTT
vgaB_227	efflux	MLSB	TAAAAGAGAATAAGGCGCAAGGA	TGTTTAGTAGCATGTTGCATTTTCC
pica_229	protection	MLSB	GCAATCGAGGCGGTGTTC	TTGCCGCAGCCAATTCA
mtrE_231	efflux	MDR	CGATGTGTCGTTTTGGAAGGT	CCTGCACCATGATTCCTCAATA
oprD_234	efflux	MDR	ATGAAGTGGAGCGCCATTG	GGCCACGGCGAACTGA
penA_236	protection	Beta-lactam	AGACGGTAACGTATAACTTTTTGAAAGA	GCGTGTAGCCGGCAATG
pmrA_239	deactivate	Other	TTTGCAGGTTTTGTTCCTAATGC	GCAGAGCCTGATTTCTCCTTTG
ttgA_243	efflux	MDR	ACGCCAATGCCAAACGATT	GTCACGGCGCAGCTTGA
ttgB_244	efflux	MDR	TCGCCCTGGATGTACACCTT	ACCATTGCCGACATCAACAAC
mepA_245	efflux	MDR	ATCGGTCGCTCTTCGTTCAC	ATAAATAGGATCGAGCTGCTGGAT
mexE_246	efflux	MDR	GGTCAGCACCGACAAGGTCTAC	AGCTCGACGTACTTGAGGAACAC
qnrA_248	protection	Fluoroquinolone	AGGATTTCTCACGCCAGGATT	CCGCTTTCAATGAAACTGCAA
lnuA_251	deactivate	MLSB	TGACGCTCAACACACTCAAAAA	TTCATGCTTAAGTTCCATACGTGAA
mtrD_253	efflux	MDR	CGGAGTCCATCGACCATTTG	ATCGTCGGCAAGGAGAATCA
vat(E)_263	deactivate	MLSB	GACCGTCCTACCAGGCGTAA	TTGGATTGCCACCGACAATT
ermY_270	protection	MLSB	TTGTCTTTGAAAGTGAAGCAACAGT	TAACGCTAGAGAACGATTTGTATTGAG
cfr_277	protection	MLSB	GCAAAATTCAGAGCAAGTTACGAA	AAAATGACTCCCAACCTGCTTTAT
sulA/folP_280	protection	Sulfonamide	CAGGCTCGTAAATTGATAGCAGAAG	CTTTCCTTGCGAATCGCTTT
ermA/ermTR_283	protection	MLSB	ACATTTTACCAAGGAACTTGTGGAA	GTGGCATGACATAAACCTTCATCA
oleC_285	efflux	MLSB	CCCGGAGTCGATGTTCGA	GCCGAAGACGTACACGAACAG
carB_288	efflux	MLSB	GGAGTGAGGCTGACCGTAGAAG	ATCGGCGAAACGCACAAA
pikR2_290	protection	MLSB	TCGTGGGCCAGGTGAAGA	TTCCCCTTGCCGGTGAA
tetE_291	efflux	Tetracycline	TTGGCGCTGTATGCAATGAT	CGACGACCTATGCGATCTGA
tetbP_294	efflux	Tetracycline	TGGGCGACAGTAGGCTTAGAA	TGACCCTACTGAAACATTAGAAATATACCT

tetT_297	protection	Tetracycline	CCATATAGAGGTTCCACCAAATCC	TGACCCTATTGGTAGTGGTTCTATTG
tolC_298	efflux	MDR	GGCCGAGAACCTGATGCA	AGACTTACGCAATTCCGGGTTA
vanRB_306	protection	Vancomycin	GCCCTGTCGGATGACGAA	TTACATAGTCGTCTGCCTCTGCAT
vanRC_307	protection	Vancomycin	TGCGGGAAAAACTGAACGA	CCCCCCATACGGTTTTGATTA
vanRC4_308	protection	Vancomycin	AGTGCTTTGGCTTATCTCGAAAA	TCCGGCAGCATCACATCTAA
vanRD_309	protection	Vancomycin	TTATAATGGCAAGGATGCACTAAAGT	CGTCTACATCCGGAAGCATGA
vanSC_311	protection	Vancomycin	ATCAACTGCGGGAGAAAAGTCT	TCCGCTGTTCCGCTTCTT
vanTE_314	protection	Vancomycin	GTGGTGCCAAGGAAGTTGCT	CGTAGCCACCGCAAAAAAAT
vanTC_315	protection	Vancomycin	ACAGTTGCCGCTGGTGAAG	CGTGGCTGGTCGATCAAAA
vanTG_316	protection	Vancomycin	CGTGTAGCCGTTCCGTTCTT	CGGCATTACAGGTATATCTGGAAA
vanYB_317	protection	Vancomycin	GGCTAAAGCGGAAGCAGAAA	GATATCCACAGCAAGACCAAGCT
vanYD_318	protection	Vancomycin	AAGGCGATACCCTGACTGTCA	ATTGCCGGACGGAAGCA
imp-marko_324	deactivate	Beta-lactam	GGAATAGAGTGGCTTAATTC	GGTTTAACAAAACAACCACC
qnrB-bob_resign_328-n	efflux	Amphenicol	GCGACGTTCAGTGGTTCAGA	GCTGCTCGCCAGTCGAA
merA-marko_331	other	MDR	GTGCCGTCCAAGATCATG	GGTGGAAGTCCAGTAGGGTGA
int1-a-marko_336	MGE	Integrase	CGAAGTCGAGGCATTTCTGTC	GCCTTCCAGAAAACCGAGGA
intI2_338	MGE	Integrase	TGCTTTTCCCACCCTTACC	GACGGCTACCCTCTGTTATCTC
IncN_rep_340	MGE	Plasmid-inc	AGTTCACCACCTACTCGCTCCG	CAAGTTCTTCTGTTGGGATTCCG
IncN_oriT_341	MGE	Plasmid-inc	TTGGGCTTCATAGTACCC	GTGTGATAGCGTGATTTATGC
IncP_oriT_342	MGE	Plasmid-inc	CAGCCTCGCAGAGCAGGAT	CAGCCGGGCAGGATAGGTGAAGT
IncQ_oriT_343	MGE	Plasmid-inc	TTCGCGCTCGTTGTTCTTCGAGC	GCCGTTAGGCCAGTTTCTCG
IncW_trwAB_344	MGE	Plasmid-inc	AGCGTATGAAGCCCGTGAAGGG	AAAGATAAGCGGCAGGACAATAACG
qacH_351_351	efflux	MDR	GTCGGTGTTGCTTATGCAGTCT	CAACCAGGCAATGGCTGTAA
marR_355	regulator	MDR	GCTGTTGATGACATTGCTCACA	CGGCGTACTGGTGAAGCTAAC
trfa_358	MGE	Transposase	ACGAAGAAATGGTTGTCCTGTTC	CGTCAGCTTGCGGTACTTCTC
intI1F165_clinical_359	MGE	Integrase	CGAACGAGTGGCGGAGGGTG	TACCCGAGAGCTTGGCACCCA
NDM new_362-n2-25-15	deactivate	Beta-lactam	GGCCACACCAGTGACAATATCA	CAGGCAGCCACCAAAAGC
sul1 NEW_363	protection	Sulfonamide	GCCGATGAGATCAGACGTATTG	CGCATAGCGCTGGGTTTC
orf37-IS26_365	MGE	Insertional	GCCGGGTTGTGCAAATAGAC	TGGCAATCTGTCGCTGCTG
orf39-IS26_366	MGE	Insertional	GCGCGTCGAGCATCAATAG	CAGTTGTGCTGCTGGTGGTC
ISPps1-pseud_369	MGE	Insertional	CACACTGCAAAAACGCATCCT	TGTCTTTGGCGTCACAGTTCTC
ISSm2-Xanthob_370	MGE	Insertional	TGGATCGACCGGTTCCAT	GCTGACCGAGCTGTCCATGT
ISAba3-Acineto_371	MGE	Insertional	TCAGAGGCAGCGGTATACGA	GGTTGATTCAGTTAAAGTACGTAAAACTTT
ISEfm1-Entero_372	MGE	Insertional	AGGTGTCCATGACGTGAAAGTG	TCCTTTGTCCCCTAGGATATTGG
mexB_374	efflux	MDR	CTGGAGATCGACGACGAGAAG	GAAATCGTTGACGTAGCTGGAA
cmlA5_375	efflux	Amphenicol	GCGCTCTTCGAGGATTCG	CCGCCCAAGCAGAAGTAGAC
IS1111_376	MGE	Insertional	GTCTTAAGGTGGGCTGCGTG	CCCCGAATCTCATTGATCAGC
PAMBL-1-F_377old_377	MGE	Plasmid-rep	CAGGCTCTTAATGTGATA	TTATGCTCAATACTCGTG
pAKD1-IncP-1__380	MGE	Plasmid-rep	GGTAAGATTACCGATAAACT	GTTCGTGAAGAAGATGTA
pBS228-IncP-1__381	MGE	Plasmid-rep	CAATCCATCGACAATCAC	GACAATCAGCTACTTCAC
IS1133_383	MGE	Insertional	GCAGCGTCGGGTTGGA	ACGCGTTCGAACAACTGTAATG
TN5_384	MGE	Insertional	CAGCATAAAAAATCCCGACAACA	CCCCGCAACAGACATACGT
aac3ia_400	deactivate	Aminoglycoside	ACGTTCTGCCAAAGTTTGAG	ACTGCCGGATCGTCAC
aph4ib_402	deactivate	Aminoglycoside	GGGAACACCGTGCTCACC	GTTGGTCCCGTGCAGGTC
aph3via_403	deactivate	Aminoglycoside	TCTCATGGCGATATCACGGATAG	TTTCCTCCGATGCATCCTCTC

aph6ic_404	deactivate	Aminoglycoside	CACGACAACGTGCTCGAC	CCGTCTTCGGCGAACCA
ArmA_405	protection	Aminoglycoside	TCTTCGACGAATGAAAGAGTCG	GCTAATGGATTGAAGCCACAACC
spcN_406	deactivate	Aminoglycoside	GCTATGTGCTGGTGGACTGG	GGAACCACTCGACGAACTCG
spec_aph_407	deactivate	Aminoglycoside	GGTGCTGATATGAATGCCTTTGG	CATTGGGCGCATCAATAAATGG
aac(3)-ib_408	deactivate	Aminoglycoside	CAGCGAGACGTTCATCGC	CACGCTTCAGGTGGCTAATC
aac(3)-id_ie_409	deactivate	Aminoglycoside	AGATAGTTATGCCCGCAACAAG	ACGCGCTGCGCCTATA
aac(3)-iid_iii_iif_iia_iie_4	deactivate	Aminoglycoside	CGATGGTCGCGGTTGGTC	TCGGCGTAGTGCAATGCG
aac(3)-xa_411	deactivate	Aminoglycoside	GCAAGCGGTTCGTGACGTA	TCAGGTGCTCCTCGATCCAG
Aac6-Aph2_412	deactivate	Aminoglycoside	CCAAGAGCAATAAGGGCATACCAA	GCCACACTATCATAACCACTACCG
aac(6)-ig_413	deactivate	Aminoglycoside	GCGATGTTAGAAGCCTCAATTCG	CACACTTCGGCCTGTCGAA
aac(6)-iic_414	deactivate	Aminoglycoside	CAGTCTTTGGCTAATCCATCACAG	AACGAACCCGGCCTTCTC
aac(6)-ij_415	deactivate	Aminoglycoside	ATGCCTGTATCTGAATCCCTGATG	GGCAATCGCTTGTTGAGTATCTG
aac(6)-im_417	deactivate	Aminoglycoside	CGTGAGCATTATACAGAGCAATGG	CCATTTCCGTTCGTAGATATTGGC
aac(6)-ir_418	deactivate	Aminoglycoside	GCTATAACGATCAGCAGCAAGC	CGCGATGCATGGCATGAC
aac(6)-is_iu_ix_419	deactivate	Aminoglycoside	AAGCTTACTCTGGCCTGATCATG	TGCCTGAACGTCGATATTCAGG
aac(6)-iv_ih_420	deactivate	Aminoglycoside	TTGGCTTATACCGACACCCA	CCCGTTGCGATACCTGAAC
aac(6)-iw_421	deactivate	Aminoglycoside	TGCGTCAGTTACTTACACGAAC	CCTGATGCATTGCATGACTGA
aac(6)-iz_422	deactivate	Aminoglycoside	TGCGCCATGACTACGTGAAC	GACTGTCCGAAGCCAGTTCG
aacA43_423	deactivate	Aminoglycoside	CTTGGCCTACATTAGATTCAGCTC	GCTCTCAATCTTTGATAGGAGCAG
aadA6_424	deactivate	Aminoglycoside	CCATCGAGCGTCATCTGGAA	CCCGTCTGGCCGGATAAC
aadA7_425	deactivate	Aminoglycoside	CACTCCGCGCCTTGGA	TGTGGCGGGCTCGAAG
aadA10_426	deactivate	Aminoglycoside	ACAGGCACTCAACGTCATCG	CGCGGAGAACTCTGCTTTGA
aadA16_427	deactivate	Aminoglycoside	ACGGTGGCCTGAAGCC	GAATTGCAGTTCCCGTCTGG
aadA17_428	deactivate	Aminoglycoside	TGTACGGCTCCGCAGTG	CACGGAATGATGTCGTCGTG
aadB_429	deactivate	Aminoglycoside	CCTGCTTGGTGGGCAGAC	CGGCACGCAAGACCTCAA
ant4-ib_430	deactivate	Aminoglycoside	GATGGCCGCTGACACATG	TCAACATTGCGCCATAGTGG
ant6-ia_431	deactivate	Aminoglycoside	TCGCCATGAGCTGCTGA	CCTATCATACTCCGGATAGGCATA
aph3-ib_432	deactivate	Aminoglycoside	AACAGGTTTGGGAGGCGATG	CGCAACAAGCCTCTCCTGAA
aph3-viia_433	deactivate	Aminoglycoside	CTCTCTCATGGAGATATGAGCGCTA	AATCCGGTTCAAGTCCCAACATG
aph4-ia_434	deactivate	Aminoglycoside	CGCTCCCGATTCCGGAA	CACAGTTTGCCAGTGATACACA
aph(3)-ia_435	deactivate	Aminoglycoside	TAACAGCGATCGCGTATTTCG	TCCGACTCGTCCAACATCAATA
apmA_436	deactivate	Aminoglycoside	GGCGCACATGCATTCATCA	CTATACTCCAGTCCCACCATTTGA
aph_viii_437	deactivate	Aminoglycoside	TCGGTATCCCGGTTGTGAG	ACACGAGGTACGGGAATCC
acc3-iva_438	deactivate	Aminoglycoside	CCAACACGACGCTGCATC	GCTGTCGCCACAATGTCG
tet40_500	efflux	Tetracycline	CTGTCCGTGCGCAATATATCC	GGATATATTGCGCACGGACAG
tetD_501	efflux	Tetracycline	AATTGCACTGCCTGCATTGC{EndPos:952}	GACAGATTGCCAGCAGCAGA{EndPos:112
tetPB_502	efflux	Tetracycline	TGGCAAGACGAGTTTGACTGA	GATCGCTCCACTTCAGCGATAA
tet39_505	efflux	Tetracycline	TATAGCGGGTCCGGTAATAGGTG	CCATAACGATCCTGCCCATAGATAAC
tetG_F_507	efflux	Tetracycline	TCGCGTTCCTGCTTGCC	CCGCGAGCGACAAACCA
tetR_506, 508	regulator	Tetracycline	CCGTCAATGCGCTGATGAC	GCCAATCCATCGACAATCACC
dfra14_600	protection	Trimethoprim	CGGATCATGTCATTGTTTCAGG	ATGTTAGAGGCGAAGTCTTGG
dfra17_601	protection	Trimethoprim	CGGGAACGGCCCTGATATTCC	CGTGTTGCGACCGCATACTTTC
dfra7_602	protection	Trimethoprim	GTAATCGGTAGTGGTCCTGA	ATCAGGACCACTACCGATTAC
dfra21_603	protection	Trimethoprim	TTGTTTCAACGCTGTCGCA	GGTTTCGGTTGAGACAAGCTC
dfra5_604	protection	Trimethoprim	CCATGGAGTGCCAAAGGTG	CACCTTTGGCACTCCATGG

dfrA8_606	protection	Trimethoprim	GGTCGCACCTGCATCGTTA	AGCGCCACCAATGACGTAG
dfrA10_607	protection	Trimethoprim	CTTCAACTATCACAGAGCACGAAG	TCTACCGGTACATACACATCAGC
dfrA15_608	protection	Trimethoprim	AGGCCGAAAGACTTTCGAGTC	TCACCTTCTGGCTCAATGTCG
dfrA18_609	protection	Trimethoprim	GGAGCGAATCAAGGAGAAAGGAA	GCAATGCGTTGATCGGTATTCTC
dfrA22_610	protection	Trimethoprim	CAGCCGAACACGGCAAAG	CGGAGTGCGTGTACGTGA
dfrA25_611	protection	Trimethoprim	TCAAACTGGACAGCGGCTA	GTCGATTGTCGACACATGCA
dfrA27_612	protection	Trimethoprim	GCCGCTCAGGATCGGTA	GTCGAGATATGTAGCGTGTCG
dfrAB4_613	protection	Trimethoprim	CGGTTCGCATTCCCATCAAA	CGCAGTCATGGATAAATCTGG
dfrC_614	protection	Trimethoprim	GTCGCTCACGATAAACAAAGAGTC	CCCTTCATGGTGAAATGAAGCTTG
dfrG_615	protection	Trimethoprim	TCAATCGGAAGAGCCTTACCTGA	TGGGCAAATACCTCATTCCATTCC
dfrK_616	protection	Trimethoprim	TGCTGCGATGGATAAGAACAG	CTTCCAGGTAATGCTCTTCCG
dfrBmulti_617	protection	Trimethoprim	ACCAAGGCAGAAGTGAAGTCA	GGTGAGCCTCAGACTCGAC
fosb_700	deactivate	Other	CTTGCAGGCCTATGGATTGC	TCTGTTCTCAAGTGTGCCAGTA
fosX_702	deactivate	Other	AGCTGGTTTGTGGATTTGCA	CCACACCGAGAGCTTTAATCCG
Arr2_703	deactivate	Other	TTGGCGATTGGTGACTTGCTAA	ATCGTCTTCGAACGGTCCTG
mcr-1_704	protection	Other	CACATCGACGGCGTATTCTG	CAACGAGCATACCGACATCG
sulIII_705	protection	Sulfonamide	CGCGCTCAAGGCAGATG	GGGAATGCCATCTGCCTTG
ere(A)_801	deactivate	MLSB	GATAATTCTGCTGGCGCACA	GCAGGCGTGGTCACAAC
ere(B)_802	deactivate	MLSB	TCGTATATGGCGGGCGTAGTA	GGTCCAAGATGGGTGAATGCA
erm(A)_803	protection	MLSB	TCGTTGAGAAGGGATTTGCGA	TTGCATGCTTCAAAGCCTGTC
erm(B)_804	protection	MLSB	GAACACTAGGGTTGTTCTTGCA	CTGGAACATCTGTGGTATGGC
erm(D)_805	protection	MLSB	TTTCCGGACAGCATTTGATGC	TCCACTGCCAATACCTTACCG
erm(E)_806	protection	MLSB	GTCACGCAGCTGGAGTTCG	CGGTGAAGCACAGCTCGAC
erm(G)_807	protection	MLSB	CCCTTGAATTAGTACAGAGGTG	GCAAACTCGTATTCCACGA
erm(O)_808	protection	MLSB	TGATGACGGCTCAGTGG	GTGCACCAGCGCCTGA
erm(Q)_809	protection	MLSB	TGAAAGCCATGCGTCTGAC	TTCAGCTGGCAGCTTAAGC
erm(S)_810	protection	MLSB	GAGTACGCCCGCAAACG	GCGTTCGATCCGGAGGA
lnuB_811	deactivate	MLSB	GGATCGTTTACCAAAGGAGAAGG	AGCATAGCCTTCGTATCAGGAA
mphA_812	deactivate	MLSB	TCAGCGGGATGATCGACTG	GAGGGCGTAGAGGGCGTA
vat(A)_813	deactivate	MLSB	ATGAACGGAGCGAATCATCGG	CCATACCGATCCAAACGTCATTTC
erm(34)_814	protection	MLSB	AAAGCGGTTTACAAGCGTTTCG	GGGTGCTCTAGGGTTGTTTAGTG
erm(35)_815	protection	MLSB	CCTTCAGTCAGAACCGGCAA	GCTGATTTGACAGTTGGTGGTG
erm(42)_816	protection	MLSB	TGTTGAGATTGGGCCTGGA	CTAAGGGTGGGTTCTCACTATCTA
erm(F)_817	protection	MLSB	TCTGATGCCCGAAATGTTCAAG	TGAAGGACAATTGAACCTCCCA
lnu(F)_818	deactivate	MLSB	ATACCGGTCATTTCCACTTGGC	GCATCAGGCTGATGAGGTTCAA
lsa(C)_819	protection	MLSB	AAACGGCGTGAAAGTATCAGG	TTGTGGTGATGTAACGGATGC
mef(B)_820	efflux	MLSB	CCGATAGGCTTACTTGTTGCAG	AGTCCACTTGCGGTTTCATTG
msr(D)_823	efflux	MLSB	GGCAAGCTAGGTGTTGAGC	ATTGCTCAACACCTAGCTTGC
msr(E)_824	efflux	MLSB	CGGCAGATGGTCTGAGCTTAAA	CGCACTCTTCCTGCATAAAGGA
vgaA_826	efflux	MLSB	GGAAGCTATAGAGGCGTTTGAATC	CCGAAGGTTCAATACTCAATCGAC
vga(A)LC_827	protection	MLSB	GTGAAGATGTCTCGGGTACAATTG	GAAATACCAGGATTCCCATGCAC
vatB_828	deactivate	MLSB	GCAATTGTTGCTGCGAATTCAG	GTGCTGACCAATCCCACCA
cat_900	deactivate	Phenicol	ATCGGCCAGACTGGATATCGA	CACAGCTCCAGTTGCAACAAC
catA2_902	deactivate	Phenicol	CCTGGAACCGCAGAGAACA	CGGAACTCCGGAAACTGATTAAC
catA3_903	deactivate	Phenicol	CTGATTGCTCAGGCCGTGAA	ATGAGTATGGGCAACTCAGTGC

catB2_904	deactivate	Phenicol	GCTACTATTCCGGCTATTACCATG	GGGCTCCTCGTTCATGTAGA
catB9_906	deactivate	Phenicol	CACCTTATGAAGTGGTCGGTTCA	GTCTGATGAACACAGAGACTGCA
cat(pC221)_908	deactivate	Phenicol	AATGACCGTATGCTGCAAGAAG	TTTGCCTGCTATGGCATTCTG
catP_909	deactivate	Phenicol	CCTTTGGACTGAGTGTAAGTCTGA	TAAAGCCATCGAAGGTTGACCA
catQ_910	deactivate	Phenicol	AGGTGCACTTACAGTATGACTGC	AACGTGGGAAGTTCTCGTCATAC
cmlV_911	deactivate	Phenicol	GCCCTCATCACCGTCTTCG	GGACGTTGGCGATGGAGAG
fexA_912	efflux	Phenicol	TGGTGTGGCTGTTGCAATCTTA	CCAAGGTACAAAGCACCTTGGA
floR_913	efflux	Amphenicol	AACCCGCCCTCTGGATCA	GCCGTCGAGAAGAAGACGAA
vanG_1002	protection	Vancomycin	TGTTTCGCAGAACCGTGTCAA	CCCTGCACTGTTCCATCTTCTC
vanC2/vanC3_1003	protection	Vancomycin	TGACTGTCGGTGCTTGTGA	GATAGAGCAGCTGAGCTTGTTC
beta_ccra_1104	deactivate	Beta-lactam	CACTGGCACGGCGATTGTA	CGGCAGCCAAACCACGATA
cefa_ampc_1105	deactivate	Beta-lactam	CAGGATCTGATGTGGGAGAACTA	TCGGGAACCATTTGTTGGC
bl1acc_1107	deactivate	Beta-lactam	TGTTATCCGTGATTACCTGTCTGG	CTCAGCGAGCCAACTTCAAATA
blaCTX-M-1,3,15_1108	deactivate	Beta-lactam	CGTACCGAGCCGACGTTAA	CAACCCAGGAAGCAGGCA
blaSHV-11_1110	deactivate	Beta-lactam	TTGACCGCTGGGAAACGG	TCCGGTCTTATCGGCGATAAAC
bl3_cpha_1113	deactivate	Beta-lactam	GTAACGCCTACTGGAAGTCCA	CAGCTTCTCCTTGAGAATGCAG
blaB-11,13,14_1114	deactivate	Beta-lactam	CGTGCCGGAGGTCTTGAATA	GGGATAGTAAACCTGAAACTCGGA
blaIND_1115	deactivate	Beta-lactam	CGCCTGTTAAACCCAACCTGTA	CGCTCTGTCATCATGAGAGTGG
blaLEN_1116	deactivate	Beta-lactam	TGTTCGCCTGTGTGTTATCTCC	GCAGCACTTTAAAGGTGCTCAC
blaOXY-1_1118	deactivate	Beta-lactam	AAAGGTGACCGCATTCGC	CCAGCGTCAGCTTGCG
bla-SME_1119	deactivate	Beta-lactam	GAGGAAGACTTTGATGGGAGGATTG	CGCTATATTGCAATGCAGCAGAAG
blaCARB_1120	deactivate	Beta-lactam	TGATTTGAGGGATACGACAACTCC	CTGTAATACTCCGAGCACCAA
blaGOB_1121	deactivate	Beta-lactam	CTTGGGCTTGAATGCTCAGGTA	TGTATGGTCGTAGTGAGCCTGA
blaHERA_1122	deactivate	Beta-lactam	GGGCAACCGCATTCTGAC	GCATCTCCCACTTTATCGTCAC
blaMIR_1123	deactivate	Beta-lactam	CGGTCTGCCGTTACAGGTG	AAAGACCCGCGTCGTCATG
blaFOXnew_1125	deactivate	Beta-lactam	CCTACGGCTATTCGAAGGAAGATAAG	CCGGATTGGCCTGGAAGC
nonmobile_blaADC_1127	deactivate	Beta-lactam	GGTATGGCTGTGGGTGTTATTCA	AGGCAAGGTTACCACTTGTATACG
nonmobile blaBEL_1128	deactivate	Beta-lactam	ATGTCCATGGCACAGACTGTG	CCTGTCTTGTCACCCGTTACC
blaIMI_1129	deactivate	Beta-lactam	ACATCTACACCTGCAGCAGTAG	AATCGCTTGGTACGCTAGCA
norA_1200	efflux	Fluoroquinolone	ATCGCCGTTTGGTGGTACG	TCCACCAATCCCTGGTCCTAAA
qepA_1_2_1201	efflux	Fluoroquinolone	GGGCATCGCGCTGTTC	GCGCATCGGTGAAGCC
qnrB4_1202	protection	Fluoroquinolone	TCACCACCCGCACCTG	GGATATCTAAATCGCCCAGTTCC
qnrS1_S3_S5_1203	protection	Fluoroquinolone	CCACTTTGATGTCGCAGATCTTC	CCCTCTCCATATTGGCATAGGAAA
qnrVC1_VC3_VC6_1204	protection	Fluoroquinolone	CTCACATCAGGACTTGCAAGAA	ATGAAGCATCTCGAAGATCAGC
qnrVC4_VC5_VC7_1205	protection	Fluoroquinolone	TTCCTTTAAACGGGCAAACCTC	CGATACCTGATTCATGAAGCTAGC
mdth_1300	efflux	MDR-chromo	ATGCTGGCTGTACAAGTGATG	CACTCCAGCGGGCGATA
cefa_qacelta_1301	efflux	MDR-mobile	TAGTTGGCGAAGTAATCGCAAC	TGCGATGCCATAACCGATTATG
mdtg_1302	efflux	MDR	TTCCAGCCGGTCAGCAA	GACATCTCCCGCGAGTTCG
pcoA_1303	deactivate	MDR-mobile	TGGCGTATGGAGTTTCAATGC	GAATAATGCCGTGCCAGTGAA
silE_1304	deactivate	MDR-mobile	GGTGGAAAGTCATCAGAGGATGA	CAAAGCCCAGCAAGGATGC
arsA_1305	efflux	MDR-mobile	CAGGTCAGCCGCATCAACC	GCCTGAAACACGGCAATTTCTTC
qacA/B_1306	efflux	MDR-mobile	AAGGGCCACTGCATTAGCTG	CCAGTCCAATCATGCCTGCA
qacF/H_1308	efflux	MDR-mobile	CTGAAGTCTAGCCATGGATTCACTAG	CAAGCAATAGCTGCCACAAGC
bacA_1500	deactivate	Other	ATCCGCGGCACCCTGA	CCTGCTTGATGGACTTGATGAAGA
aac(6)I1_1501	deactivate	Aminoglycoside	GGGAATTATCGGAATAGCTCTTGG	TTGGGCTGTTCTTCCTAGCTAA

aac(6)-ly_1502	deactivate	Aminoglycoside	GCCTCAATCCGCCACGATTA	ACGCGCTCTGTTTCCTCAAA
aph6ia_1503	deactivate	Aminoglycoside	CGCTGGGAGCTGAAGAGG	AGCATCGTGCTGCTCTCC
bexA/norM_1504	efflux	MDR	TCGGGCATCCCGTTTATGATC	GTAGGCTGCGCATAATACCCA
ampC_1505	deactivate	Beta-lactam	CTGGCGCATACCTGGATTAC	GCCAGTTCAGCATCTCCCA
blaOXA10_1506	deactivate	Beta-lactam	CGACCGAGTATGTACCTGCTTC	TCAAGTCCAATACGACGAGCTA
tetPA_1507	efflux	Tetracycline	GGAAACCTTAGTTCAGTGACTTGG	CCCATTTAACCACGCACTGAA
blaIMIR_1508	regulator	Beta-lactam	AGCCGGACTAGAGCTTCATG	GGCAGAACTCATCATCTGCAAA
mdtA_1509	efflux	MDR	ACAAGCCCAGGGCCAAC	CCTTAATGGTGCCTTCGGTTTC
aac3-Via_1510	deactivate	Aminoglycoside	GTGTCCGTCGCCAAGGA	GGTGACGGCCTTGTCGA
mefA_1511	efflux	MLSB	TAATTATCGCAGCAGCTGGTTC	GTTCCCAAACGGAGTATAAGAGTG
blaTEM_1512	deactivate	Beta-lactam	CGCCGCATACACTATTCTCAG	GCTTCATTCAGCTCCGGTTC
tetM_1513	protection	Tetracycline	GGAGCGATTACAGAATTAGGAAGC	TCCATATGTCCTGGCGTGTC
vanA_1514	protection	Vancomycin	GGGCTGTGAGGTCGGTTG	TTCAGTACAATGCGGCCGTTA
vanXA_1515	protection	Vancomycin	TCGTTGGGACGCTAAATATGC	GGACGGTAACCGTCCCATA
tetJ_1516	efflux	Tetracycline	CAGCGCCCATACGCCATTTA	CCTACTTCAGTAGTGTGCCAAGC
blaPER_1517	deactivate	Beta-lactam	GCAAATGAAGCGCAGATGC	GACCACAGTACCAGCTGGTA
tet(38)_1518	efflux	Tetracycline	AAGCGACATTAGCCGGTTTAG	CTGCTCGTACTTAAGCCAAGG
lnuC_1519	deactivate	MLSB	GGGTGTAGATGCTCTTCTTGGA	CTTTACCCGAAAGAGTTTCTACCG
fabK_1520	protection	Other	CAGGAGCAGGAAATCCAAGC	CCAGCTTCCATTCCTTCTGC
vanSB_1521	protection	Vancomycin	GAAGATAAAGAGGGAAGCGTACTC	CCGAATTGTCAGCCCTTGATAA
intI3_1522	MGE	Integrase	CAGGTGCTGGGCATGGA	CCTGGGCAGCATCACCA
KPC_1523	deactivate	Beta-lactam	GCCGCCAATTTGTTGCTGAA	GCCGGTCGTGTTTCCCTTT
mobA_1524	mobA	Plasmid	GCTTCCCGTAACGAGGTAGT	CCTTGAACGGTATCAGCACG
traN_1525	MGE	Plasmid	GCTTGGCGGTCAGCAATT	TTAGGAATAACAATCGCTACACCTTTA
tra-A_1526	MGE	Plasmid	AAGTGTTCAGGGTGCTTCTGCGC	GTCATGTACATGATGACCAAAA
trb-C_1527	MGE	Plasmid	CGGYATWCCGSCSACRCTGCG	GCCACCTGYSBGCAGTCMCC
ISCR1_1528	MGE	Insertional	ATGGTTTCATGCGGGTT	CTGAGGGTGTGAGCGAG
copA_1529	other	MDR-mobile	TGCACCTGACVGGSCAYAT	GVACTTCRCGGAACATRCC
Staphylococci_1532 (mec	other	Taxonomic	CGCAACGTTCAATTTAATTTTGTTAA	TGGTCTTTCTGCATTCCTGGA
A.baumannii_1535 (ompA	other	Taxonomic	TCTTGGTGGTCACTTGAAGC	ACTCTTGTGGTTGTGGAGCA
czcA_1536	efflux	MDR-mobile	GCCTTGTTCATCGGCGAAC	GGCAATGTCGCCTTCGTTC
optrA_1538	protection	Phenicol	GGTGGATGAAGTCCGTACGG	AGGTTAGACCTCCAAGAGCCA
tet44_1539	protection	Tetracycline	CTCATGTAGATGCAGGAAAGACG	GTAACTGCTGCCTGAATTGTGA
aph3-III_1540	deactivate	Aminoglycoside	CAGAAGGCAATGTCATACCACTTG	GACAGCCGCTTAGCCGAA
ant6-ib_1541	deactivate	Aminoglycoside	AGAACATCCGACAGCACGTTC	CCAACCTTCCATGAAATCATTCGC
ARR-3_1542	deactivate	Other	GATCGTCTTCGAACGGTCCTG	TTTGGCGATTGGTGACTTGCT
mcr-2_1543	protection	Other	CGGCGTACTTTAAGCGTTATGATG	GCATTTGGCATACCATGCAGATAG
bla-ACT_1544	deactivate	Beta-lactam	AAGCCGCTCAAGCTGGA	GCCATATCCTGCACGTTGG
aac(3)-Xa_1545	deactivate	Aminoglycoside	TGTACGGCTCCGCAGTG	CACGGAATGATGTCGTCGTG
IS26_1546	MGE	Insertional	ATGGATGAAACCTACGTGAAGGTC	CGGTACTTAATCTGTCGGTGTTCA
IS3_1547	MGE	Insertional	CGGTCTGAGCTTCGGGAA	AGAACTGTCACTCCGGTCTG
IS256_1548	MGE	Insertional	CTTGCGCATCATTGGATGATGG	AAGAACGGCTCCAATTAAGCGA
sugE_1549	efflux	MDR-mobile	CTTAGTTATTGCTGGTCTGCTGGA	GCATCGGGTTAGCGGACTC
ISEcp1_1550	MGE	Insertional	CATGCTCTGCGGTCACTTC	GACGCACCTTCTTGATGACC
IS200_1551	MGE	Insertional	CCAAATACCGAAGACAAGCGTTC	CCAAACTGCTCGTAAAGCATCAG

IS1247_1552	MGE	Insertional	CGGCCGTCACTGACCAA	TCGGCAGGTTGGTGACG
IS630_1553	MGE	Insertional	CCGCCACCAGTGTGATGG	TTGGCGCTGACTGGATGC
TN5403_1556	MGE	Transposase	AAGCGAATGGCGCGAAC	CGCGCAGGGTAAACTGC
IS200_1557	MGE	Insertional	GCACACCCGATGGAACTGTAAA	TCGGCGGGATCTCCAGAAG
IS21-ISAs29_1558	MGE	Insertional	GGTCCGTCAGGCACAAGTC	GGGATCGTATCGGCAAGCC
Tn3_1559	MGE	Transposase	GCTGAGGTGTTCAGCTACATCC	GCTGAGGTAGTCACAGGCATTC
IS6/257_1560	MGE	Insertional	ATATCGTGCCATTGATGCAGAG	ACCATTGCTACCTTCGTTGAAG
IS6100_1561	MGE	Insertional	CGCACCGGCTTGATCAGTA	CTGCCACGCTCAATACCGA
IS15DI_1562	MGE	Insertional	CAATACCTTTGATGGTGGCGTAAG	CTTACGCCACCATCAAAGGTATTG
IncN_korA_1563	MGE	Plasmid-inc	GGAACGTTTGTAYCTTGTATTG	ACTCACTATCTTCTGTTGATTG
IncF_FIC_1564	MGE	Plasmid-inc	GTGAACTGGCAGATGAGGAAGG	TTCTCCTCGTCGCCAAACTAGAT
IncI1_repI1_1565	MGE	Plasmid-inc	CGAAAGCCGGACGGCAGAA	TCGTCGTTCCGCCAAGTTCGT
IncHI2-smr0018_1566	MGE	Plasmid-inc	ATAATGATTCACCGGGGTAG	CTTCAGGCTATCGTTTCG
IS91_1567	MGE	Insertional	GGATGCCACTGCTGGTCA	ACAGTGGATACAGTATCTGCTGAG
IS5/IS1182_1568	MGE	Insertional	TTCTCGAAGAATCGCCATGGC	GCTTTGGATCGCTCCAATCGA
cro_1569	MGE	Other	AGATGTTATCGACCACTTCGGA	CCGCTTGGCGATAAGCG
EAE_05855_1570	MGE	Other	CCCATCACCGCTGAACTGG	TGGGCGCTGCCATCTAAAC
tcrB_1571	efflux	MDR-mobile	GTGCCGGAACTCAAGTAGCA	GCACCGACTGCTGGACTTAA
terW_1572	other	MDR-mobile	TCAAAGAGCTACGCGAGTCATA	CCTTCCCTGTGGACTCACC
pbrT_1573	efflux	MDR-mobile	GATGCGCACTGGGCTTG	TCGGAATATGCGGAAATGCG
qnrD_1574	protection	Fluoroquinolone	CGCTGGAATGGCACTGTGA	GCTCTCCATCCAACTTCACTCC
cadC_1575	other	MDR-mobile	CGCTCTGTGTCAGGATGAAGAG	CTTTCTTATGTGCTAGGGCGATCA
qnrS2_1576	protection	Fluoroquinolone	TCCCGAGCAAACTTTGCCAA	GGTGAGTCCCTATCCAGCGA
oqxA_1577	other	Fluoroquinolone	GAGTCAACCTACCTCCACTATCA	GCTGCGAGTTATCCAGCAG
adeI_1578	efflux	MDR	CAGTCTGGTTTGCAGTAACCA	CACTCCTACAACAACAGGCAA
qnrB46,47,48_1579	protection	Fluoroquinolone	CGACGTTCAGTGGTTCAGATCTC	GCCAAGCCGCTCCATGAG

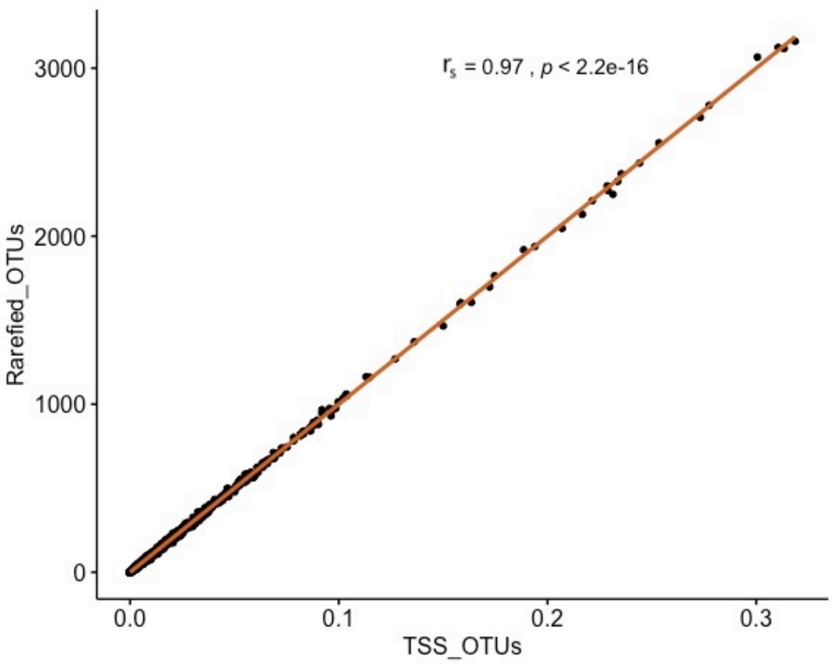

Fig. S1 Correlation between rarefied and subsampled OTUs and TSS normalized OTUs.

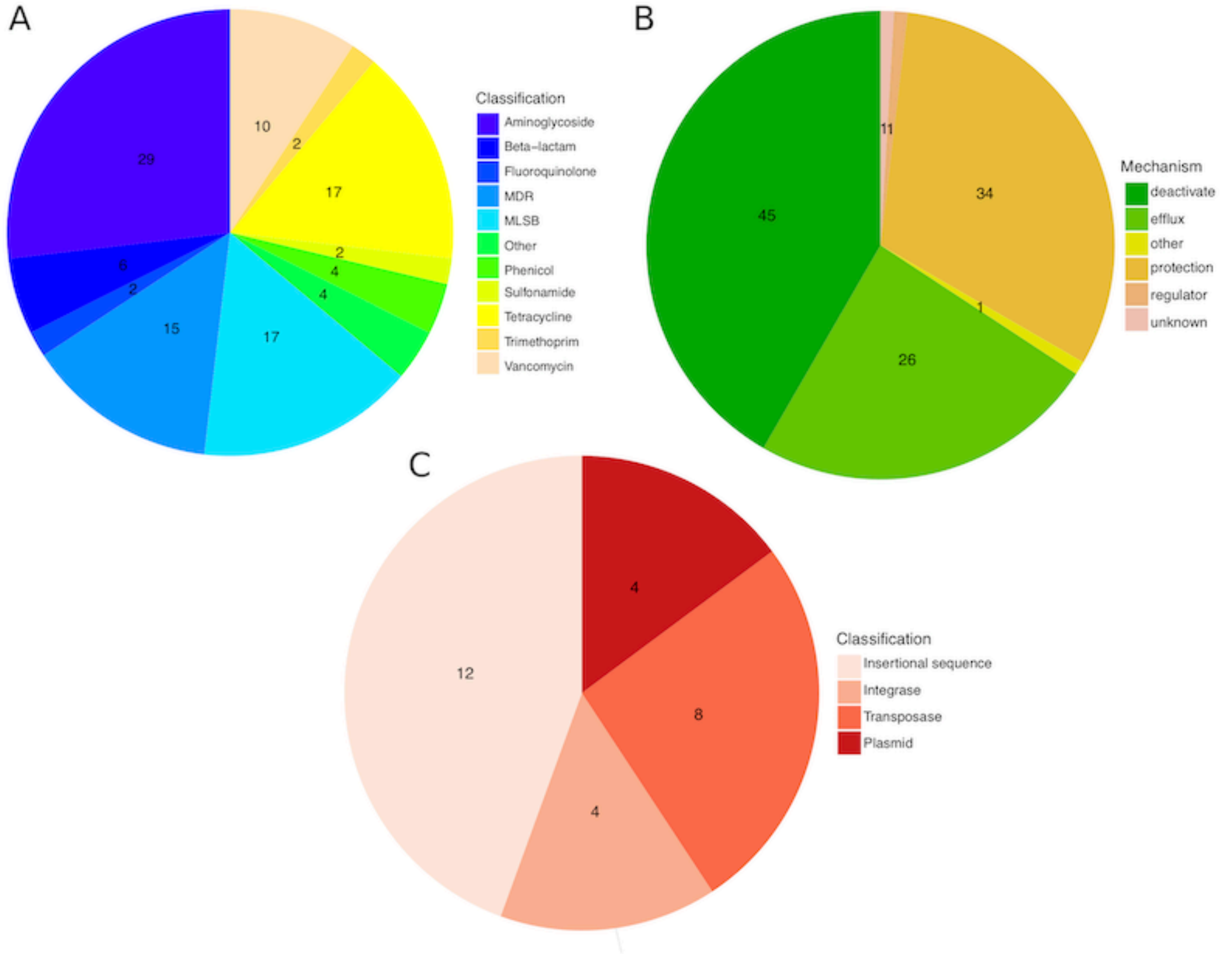

Fig. S2 Composition of positive assays grouped by (A) antibiotic group the targeted gene confers resistance, (B) resistance mechanism and (C) mobile genetic element group.

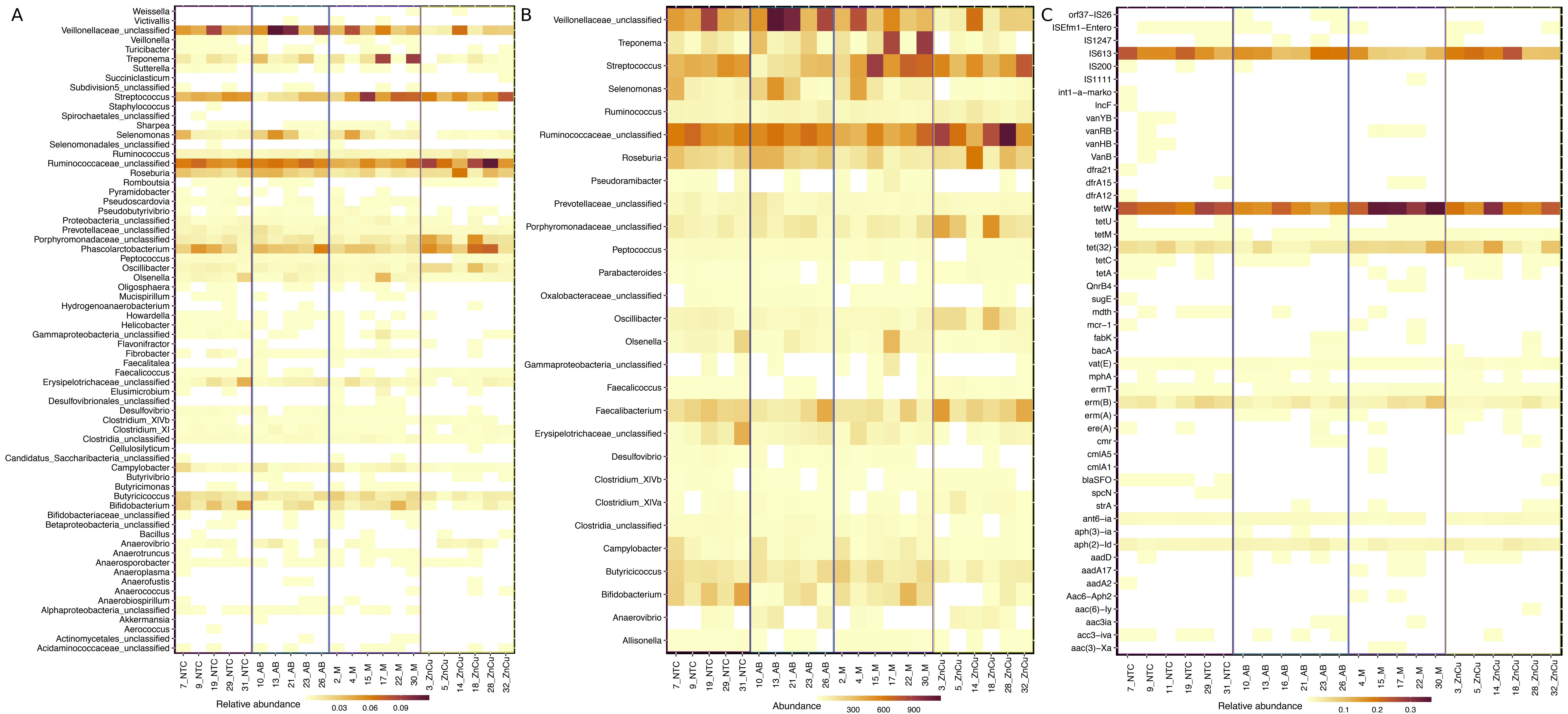

Fig. S3. Differentially abundant genera and genes. Samples on x-axis are grouped according to the treatments. Sample names are as follows: NTC= Non-treatment control, AB= Carbadox (antibiotic), M= Mushroom powder and ZnCu= zinc oxide and copper sulfate. The number in front of the group code denotes the number of the pen. Each row represents abundance of each genus or the results of each primer set (assay) (Supplementary Table S1) displayed on the y-axis. Only genera and genes with statistically significant differences between treatment groups are shown (A) Differentially abundant genera with TSS normalization. See Supplementary Table S2 for fold changes. (B) Differentially abundant genera with Rarefying and subsampling. See Supplementary Table S3 for fold changes. (C) Differentially abundant ARGs and MGEs. See Supplementary Table S4 for fold changes

Table S2. Pairwise comparisons of gamma distribution GLMs of relative abundances of each genera between treatment groups. TSS normalized OTU table was used as the input.

Comparison (X_1 - X_2)	Genus	Delta Estimate (X1 - X2) [a]	Std. Error	z-value	p.adjusted	Fold Change (X1 / X2) [b]
AB - ZnCu	Veillonellaceae_unclassified	1.21	0.39	3.14	9.43E-03	3.352
AB - ZnCu	Oscillibacter	-1.32	0.33	-3.99	3.75E-04	0.267
AB - ZnCu	Porphyromonadaceae_unclassified	-0.98	0.34	-2.93	1.79E-02	0.374
AB - ZnCu	Streptococcus	-0.93	0.25	-3.69	1.28E-03	0.396
AB - ZnCu	Selenomonas	3.04	0.68	4.46	3.67E-05	21.000
AB - ZnCu	Clostridia_unclassified	1.70	0.47	3.62	1.59E-03	5.458
AB - ZnCu	Campylobacter	1.97	0.75	2.63	4.29E-02	7.188
AB - ZnCu	Desulfovibrio	4.34	0.90	4.84	5.51E-06	76.588
AB - ZnCu	Subdivision5_unclassified	5.75	0.84	6.88	1.31E-11	313.962
AB - ZnCu	Elusimicrobium	-5.86	1.15	-5.09	1.26E-06	0.003
AB - ZnCu	Fibrobacter	4.19	0.96	4.38	8.57E-05	66.003
AB - ZnCu	Pseudoscardovia	7.72	0.55	13.91	0.00E+00	2252.752
AB - ZnCu	Alphaproteobacteria_unclassified	8.00	0.70	11.46	0.00E+00	2966.181
AB - ZnCu	Butyricimonas	5.48	0.63	8.76	0.00E+00	240.090
AB - ZnCu	Helicobacter	6.18	0.73	8.50	0.00E+00	485.265
AB - ZnCu	Sharpea	5.91	0.77	7.68	3.90E-14	368.828
AB - ZnCu	Bifidobacteriaceae_unclassified	3.67	0.90	4.09	2.24E-04	39.081
AB - ZnCu	Oligosphaera	4.87	0.64	7.58	7.14E-14	129.672
AB - ZnCu	Veillonella	5.69	0.75	7.55	9.17E-14	295.335
AB - ZnCu	Anaerotruncus	3.06	1.09	2.80	2.60E-02	21.242
AB - ZnCu	Mucispirillum	5.58	0.96	5.80	6.70E-08	264.733
AB - ZnCu	Betaproteobacteria_unclassified	3.67	0.90	4.08	2.49E-04	39.081
AB - ZnCu	Staphylococcus	-4.44	0.81	-5.48	1.53E-07	0.012
AB - ZnCu	Anaerobiospirillum	5.40	1.08	5.01	2.36E-06	221.168
AB - ZnCu	Anaerococcus	-4.10	0.94	-4.38	6.75E-05	0.017
AB - ZnCu	Faecalitalea	3.52	1.15	3.05	1.23E-02	33.792
AB - ZnCu	Turicibacter	2.41	0.87	2.76	2.92E-02	11.122
AB - ZnCu	Cellulosilyticum	-4.90	0.78	-6.31	1.31E-09	0.007
AB - ZnCu	Weissella	-4.72	1.08	-4.36	8.08E-05	0.009
AB - ZnCu	Actinomycetales_unclassified	3.83	0.80	4.81	8.08E-06	46.196
AB - ZnCu	Victivallis	3.67	0.83	4.43	6.70E-05	39.081
AB - ZnCu	Bacillus	-4.96	1.12	-4.41	6.00E-05	0.007
AB - ZnCu	Succiniclasticum	-6.25	0.78	-8.01	2.22E-15	0.002
AB - ZnCu	Pyramidobacter	5.42	0.79	6.85	8.72E-11	224.996
AB - ZnCu	Akkermansia	5.70	0.64	8.95	0.00E+00	297.725
M - AB	Roseburia	-0.86	0.29	-2.93	1.74E-02	0.425
M - AB	Streptococcus	1.06	0.25	4.21	1.86E-04	2.878

M - AB	Prevotellaceae_unclassified	-1.08	0.34	-3.19	7.44E-03	0.340
M - AB	Elusimicrobium	8.62	1.15	7.49	1.45E-13	5531.182
M - AB	Anaerovibrio	-2.73	0.90	-3.02	1.32E-02	0.065
M - AB	Clostridium_XI	-1.63	0.62	-2.62	4.38E-02	0.195
M - AB	Helicobacter	-2.15	0.73	-2.95	1.69E-02	0.117
M - AB	Gammaproteobacteria_unclassified	3.23	1.13	2.85	2.26E-02	25.292
M - AB	Bifidobacteriaceae_unclassified	2.47	0.90	2.76	2.92E-02	11.871
M - AB	Veillonella	1.94	0.75	2.58	4.85E-02	6.982
M - AB	Sutterella	-2.04	0.61	-3.33	4.81E-03	0.130
M - AB	Selenomonadales_unclassified	2.91	0.97	2.99	1.48E-02	18.286
M - AB	Anaerococcus	3.43	0.94	3.66	1.39E-03	30.910
M - AB	Anaerofustis	-4.43	0.87	-5.11	2.40E-06	0.012
M - AB	Hydrogenoanaerobacterium	-3.83	1.17	-3.27	5.95E-03	0.022
M - AB	Desulfovibrionales_unclassified	4.35	0.37	11.86	0.00E+00	77.587
M - AB	Candidatus_Saccharibacteria_unclassified	4.10	0.84	4.90	3.95E-06	60.265
M - AB	Weissella	3.43	1.08	3.17	8.20E-03	30.910
M - AB	Romboutsia	-5.31	0.50	-10.59	0.00E+00	0.005
M - AB	Bacillus	3.44	1.12	3.05	1.20E-02	31.039
M - AB	Anaeroplasma	4.51	1.00	4.52	4.88E-05	91.064
M - AB	Akkermansia	-5.70	0.64	-8.95	0.00E+00	0.003
M - NTC	Butyricimonas	1.65	0.63	2.64	4.05E-02	5.224
M - NTC	Helicobacter	-3.20	0.73	-4.39	9.84E-05	0.041
M - NTC	Sutterella	-1.64	0.61	-2.67	3.78E-02	0.194
M - NTC	Staphylococcus	-4.74	0.81	-5.86	1.45E-08	0.009
M - NTC	Anaerococcus	3.43	0.94	3.66	1.53E-03	30.910
M - NTC	Flavonifractor	4.40	1.01	4.35	7.67E-05	81.446
M - NTC	Hydrogenoanaerobacterium	-3.67	1.17	-3.13	9.69E-03	0.026
M - NTC	Desulfovibrionales_unclassified	4.35	0.37	11.86	0.00E+00	77.587
M - NTC	Butyrivibrio	3.44	1.09	3.14	8.73E-03	31.039
M - NTC	Weissella	3.43	1.08	3.17	8.66E-03	30.910
M - NTC	Actinomycetales_unclassified	3.95	0.80	4.95	3.51E-06	51.817
M - NTC	Romboutsia	-5.58	0.50	-11.13	0.00E+00	0.004
M - NTC	Spirochaetales_unclassified	-4.29	0.63	-6.81	2.92E-11	0.014
M - NTC	Pseudobutyrivibrio	2.55	0.92	2.78	2.82E-02	12.769
M - NTC	Aerococcus	-3.37	0.62	-5.47	1.80E-07	0.034
M - NTC	Anaerosporobacter	-1.46	0.56	-2.63	4.26E-02	0.231
M - NTC	Victivallis	4.10	0.83	4.95	2.80E-06	60.337
M - ZnCu	Ruminococcaceae_unclassified	-0.41	0.14	-2.94	1.73E-02	0.662
M - ZnCu	Roseburia	-0.77	0.28	-2.76	2.96E-02	0.464

M - ZnCu	Phascolarctobacterium	-0.76	0.28	-2.75	3.06E-02	0.467
M - ZnCu	Treponema	2.59	0.67	3.90	5.49E-04	13.396
M - ZnCu	Oscillibacter	-1.48	0.32	-4.67	1.37E-05	0.229
M - ZnCu	Porphyromonadaceae_unclassified	-1.10	0.32	-3.43	3.69E-03	0.334
M - ZnCu	Olsenella	1.97	0.70	2.81	2.58E-02	7.198
M - ZnCu	Butyricicoccus	0.74	0.25	2.96	1.60E-02	2.094
M - ZnCu	Ruminococcus	-0.52	0.20	-2.68	3.68E-02	0.592
M - ZnCu	Bifidobacterium	2.14	0.71	3.02	1.35E-02	8.519
M - ZnCu	Selenomonas	2.64	0.65	4.06	3.01E-04	14.018
M - ZnCu	Clostridia_unclassified	1.95	0.45	4.37	6.93E-05	7.048
M - ZnCu	Campylobacter	2.37	0.72	3.31	5.13E-03	10.670
M - ZnCu	Proteobacteria_unclassified	1.44	0.55	2.61	4.50E-02	4.204
M - ZnCu	Desulfovibrio	3.48	0.85	4.07	2.94E-04	32.331
M - ZnCu	Clostridium_XlVb	-1.53	0.44	-3.50	2.63E-03	0.217
M - ZnCu	Subdivision5_unclassified	6.72	0.80	8.43	0.00E+00	825.986
M - ZnCu	Anaerovibrio	-2.38	0.86	-2.76	2.95E-02	0.093
M - ZnCu	Fibrobacter	4.24	0.91	4.66	1.29E-05	69.651
M - ZnCu	Pseudoscardovia	8.59	0.53	16.24	0.00E+00	5400.143
M - ZnCu	Alphaproteobacteria_unclassified	7.77	0.67	11.67	0.00E+00	2357.536
M - ZnCu	Butyricimonas	6.47	0.60	10.85	0.00E+00	647.544
M - ZnCu	Acidaminococcaceae_unclassified	2.46	0.91	2.71	3.40E-02	11.649
M - ZnCu	Helicobacter	4.04	0.69	5.82	1.94E-08	56.681
M - ZnCu	Gammaproteobacteria_unclassified	3.45	1.08	3.19	7.56E-03	31.366
M - ZnCu	Sharpea	7.36	0.73	10.03	0.00E+00	1569.047
M - ZnCu	Bifidobacteriaceae_unclassified	6.14	0.85	7.19	1.39E-12	463.919
M - ZnCu	Oligosphaera	6.38	0.61	10.43	0.00E+00	590.861
M - ZnCu	Veillonella	7.63	0.72	10.63	0.00E+00	2062.008
M - ZnCu	Anaerotruncus	3.86	1.04	3.71	1.11E-03	47.385
M - ZnCu	Mucispirillum	4.16	0.92	4.54	5.59E-05	63.958
M - ZnCu	Betaproteobacteria_unclassified	4.73	0.86	5.51	1.60E-07	113.178
M - ZnCu	Staphylococcus	-4.44	0.77	-5.75	8.59E-08	0.012
M - ZnCu	Selenomonadales_unclassified	2.91	0.93	3.14	9.28E-03	18.286
M - ZnCu	Anaerobiospirillum	5.48	1.03	5.33	3.25E-07	239.354
M - ZnCu	Faecalitalea	2.91	1.10	2.64	4.10E-02	18.286
M - ZnCu	Anaerofustis	-4.90	0.83	-5.92	1.10E-08	0.007
M - ZnCu	Hydrogenoanaerobacterium	-4.43	1.12	-3.97	4.30E-04	0.012
M - ZnCu	Desulfovibrionales_unclassified	4.35	0.35	12.44	0.00E+00	77.587
M - ZnCu	Turicibacter	3.15	0.83	3.78	9.22E-04	23.243
M - ZnCu	Candidatus_Saccharibacteria_unclassified	4.10	0.80	5.14	1.24E-06	60.265

M - ZnCu	Cellulosilyticum	-4.90	0.74	-6.62	8.13E-11	0.007
M - ZnCu	Actinomycetales_unclassified	3.95	0.76	5.19	7.66E-07	51.817
M - ZnCu	Romboutsia	-6.06	0.48	-12.67	0.00E+00	0.002
M - ZnCu	Pseudobutyrivibrio	3.18	0.87	3.63	1.60E-03	23.927
M - ZnCu	Victivallis	4.10	0.79	5.20	1.13E-06	60.337
M - ZnCu	Faecalicoccus	-2.74	0.67	-4.07	2.64E-04	0.065
M - ZnCu	Succiniclasticum	-6.25	0.74	-8.41	0.00E+00	0.002
M - ZnCu	Pyramidobacter	5.15	0.75	6.83	1.90E-11	172.365
M - ZnCu	Anaeroplasma	4.51	0.95	4.74	1.23E-05	91.064
NTC - AB	Streptococcus	0.73	0.26	2.79	2.70E-02	2.077
NTC - AB	Elusimicrobium	7.41	1.20	6.16	3.36E-09	1647.648
NTC - AB	Staphylococcus	4.74	0.84	5.61	1.04E-07	114.191
NTC - AB	Selenomonadales_unclassified	4.45	1.01	4.39	5.61E-05	85.843
NTC - AB	Flavonifractor	-5.70	1.06	-5.40	1.73E-06	0.003
NTC - AB	Anaerofustis	-4.43	0.91	-4.89	3.68E-06	0.012
NTC - AB	Candidatus_Saccharibacteria_unclassified	4.88	0.87	5.59	8.49E-08	132.086
NTC - AB	Butyrivibrio	-6.22	1.14	-5.45	3.37E-07	0.002
NTC - AB	Actinomycetales_unclassified	-3.83	0.83	-4.60	1.91E-05	0.022
NTC - AB	Spirochaetales_unclassified	4.29	0.66	6.52	1.57E-10	72.866
NTC - AB	Aerococcus	3.37	0.64	5.24	5.81E-07	29.148
NTC - AB	Victivallis	-3.67	0.86	-4.24	1.26E-04	0.026
NTC - AB	Bacillus	4.29	1.17	3.65	1.46E-03	72.866
NTC - AB	Anaeroplasma	4.48	1.04	4.30	8.55E-05	88.324
NTC - AB	Akkermansia	-5.70	0.66	-8.57	0.00E+00	0.003
NTC - ZnCu	Oscillibacter	-1.13	0.33	-3.42	3.53E-03	0.323
NTC - ZnCu	Porphyromonadaceae_unclassified	-1.22	0.34	-3.65	1.45E-03	0.294
NTC - ZnCu	Bifidobacterium	2.53	0.74	3.40	3.92E-03	12.507
NTC - ZnCu	Selenomonas	2.44	0.68	3.57	1.91E-03	11.452
NTC - ZnCu	Clostridia_unclassified	1.73	0.47	3.68	1.27E-03	5.630
NTC - ZnCu	Erysipelotrichaceae_unclassified	1.73	0.58	3.00	1.44E-02	5.622
NTC - ZnCu	Campylobacter	2.30	0.75	3.06	1.17E-02	9.975
NTC - ZnCu	Desulfovibrio	4.48	0.90	5.00	5.72E-06	88.435
NTC - ZnCu	Subdivision5_unclassified	4.80	0.84	5.74	7.82E-08	120.998
NTC - ZnCu	Fibrobacter	5.10	0.96	5.33	4.06E-07	163.736
NTC - ZnCu	Pseudoscardovia	8.68	0.55	15.64	0.00E+00	5866.004
NTC - ZnCu	Alphaproteobacteria_unclassified	7.55	0.70	10.82	0.00E+00	1898.769
NTC - ZnCu	Butyricimonas	4.82	0.63	7.71	2.66E-14	123.961
NTC - ZnCu	Acidaminococcaceae_unclassified	2.47	0.95	2.60	4.59E-02	11.871
NTC - ZnCu	Peptococcus	0.98	0.32	3.05	1.25E-02	2.653

NTC - ZnCu	Helicobacter	7.24	0.73	9.94	0.00E+00	1388.032
NTC - ZnCu	Sharpea	6.53	0.77	8.49	0.00E+00	686.438
NTC - ZnCu	Bifidobacteriaceae_unclassified	5.70	0.90	6.36	5.53E-10	298.908
NTC - ZnCu	Oligosphaera	5.28	0.64	8.22	6.66E-16	195.871
NTC - ZnCu	Veillonella	6.38	0.75	8.47	0.00E+00	589.486
NTC - ZnCu	Mucispirillum	5.87	0.96	6.11	4.35E-09	355.331
NTC - ZnCu	Betaproteobacteria_unclassified	5.15	0.90	5.73	4.30E-08	172.250
NTC - ZnCu	Selenomonadales_unclassified	4.45	0.97	4.58	2.49E-05	85.843
NTC - ZnCu	Anaerobiospirillum	4.48	1.08	4.15	1.71E-04	88.324
NTC - ZnCu	Anaerococcus	-4.10	0.94	-4.38	7.07E-05	0.017
NTC - ZnCu	Faecalitalea	4.95	1.15	4.29	1.31E-04	140.592
NTC - ZnCu	Howardella	2.56	0.96	2.66	3.89E-02	12.885
NTC - ZnCu	Flavonifractor	-5.57	1.01	-5.51	3.12E-07	0.004
NTC - ZnCu	Anaerofustis	-4.90	0.87	-5.65	4.82E-08	0.007
NTC - ZnCu	Turicibacter	2.46	0.87	2.82	2.47E-02	11.715
NTC - ZnCu	Candidatus_Saccharibacteria_unclassified	4.88	0.84	5.84	1.92E-08	132.086
NTC - ZnCu	Butyrivibrio	-6.05	1.09	-5.54	1.28E-07	0.002
NTC - ZnCu	Cellulosilyticum	-4.90	0.78	-6.31	2.06E-09	0.007
NTC - ZnCu	Weissella	-4.72	1.08	-4.36	9.94E-05	0.009
NTC - ZnCu	Spirochaetales_unclassified	4.29	0.63	6.81	5.62E-11	72.866
NTC - ZnCu	Aerococcus	3.37	0.62	5.47	1.47E-07	29.148
NTC - ZnCu	Faecalicoccus	-2.21	0.71	-3.13	9.52E-03	0.110
NTC - ZnCu	Succiniclasticum	-6.25	0.78	-8.01	2.33E-15	0.002
NTC - ZnCu	Pyramidobacter	5.64	0.79	7.13	2.17E-12	282.478
NTC - ZnCu	Anaeroplasma	4.48	1.00	4.49	3.14E-05	88.324

a) Delta Estimates were calculated using glht function in the multcomp package in R. The glht function calculates the estimates by subtracting the estimate of X2 (second treatment group in the column “Comparison”) from the estimate of X1 (first treatment group in the column “Comparison”). Estimates for each treatment group were obtained with Gamma glm models, which were used as the input for the glht function. See the provided R-script (electronic supplemental material) for the procedure.

b) Fold Changes were calculated by taking the exponential function from the Delta Estimate (due to the logarithmic link function that was used in with Gamma glm models). This was done in R using the command exp(Delta.Estimate). See the provided R-script (electronic supplemental material) for the procedure. Relative abundances of all genera are presented in Supplemental figure **S3A**

Table S3. Pairwise comparisons of negative binomial GLMs of abundances of each genera between treatment groups. Rarefied and subsampled OTU table was used as the input.

Comparison (X_1 - X_2)	Genus	Delta Estimate (X1 - X2) [a]	Std. Error	z-value	p.adjusted	Fold Change (X1 / X2) [b]
AB - ZnCu	Veillonellaceae_unclassified	1.21234387	0.33269944	3.64396126	0.00158131	3.36135402
AB - ZnCu	Oscillibacter	-1.432626	0.35487338	-4.0370062	0.00028535	0.23868132
AB - ZnCu	Porphyromonadaceae_unclassified	-0.9640726	0.34852224	-2.7661723	0.02893672	0.3813367
AB - ZnCu	Streptococcus	-0.9078819	0.25697297	-3.532986	0.00224082	0.40337772
AB - ZnCu	Prevotellaceae_unclassified	0.85321027	0.27317443	3.12331678	0.00965566	2.34716981
AB - ZnCu	Selenomonas	3.04217777	0.62717819	4.85057964	5.98E-06	20.9508197
AB - ZnCu	Clostridia_unclassified	1.58321472	0.45087436	3.5114321	0.00265948	4.87058824
AB - ZnCu	Campylobacter	2.1701959	0.63654643	3.40932851	0.00353628	8.76
AB - ZnCu	Desulfovibrio	3.07269331	0.87581187	3.50839423	0.0023786	21.6
AB - ZnCu	Anaerovibrio	0.44561218	0.10322547	4.31688194	8.89E-05	1.56144578
AB - ZnCu	Peptococcus	0.74193734	0.28442987	2.6085071	0.04490273	2.1
AB - ZnCu	Oxalobacteraceae_unclassified	1.53686722	0.44070683	3.48727797	0.00260496	4.65
AB - ZnCu	Faecalicoccus	-2.3232044	0.61879187	-3.7544197	0.00089624	0.09795918
M - AB	Roseburia	-0.8614451	0.26223116	-3.2850602	0.00542459	0.422551
M - AB	Streptococcus	1.04825084	0.25689019	4.08054053	0.00028992	2.852657
M - AB	Prevotellaceae_unclassified	-1.1685243	0.27740881	-4.2122825	0.0001068	0.31082529
M - AB	Anaerovibrio	-2.7243866	0.25190262	-10.815237	0	0.06558642
M - AB	Pseudoramibacter	2.89958841	1.10369578	2.62716273	0.04261924	18.1666667
M - AB	Allisonella	1.45990618	0.53051035	2.75189011	0.02926791	4.30555556
M - AB	Oxalobacteraceae_unclassified	-1.3137237	0.41136664	-3.1935591	0.00773409	0.2688172
M - NTC	Prevotellaceae_unclassified	-0.8797698	0.27934219	-3.1494341	0.00876736	0.4148784
M - NTC	Anaerovibrio	-0.7830954	0.30180021	-2.5947478	0.04116923	0.45698925
M - ZnCu	Ruminococcaceae_unclassified	-0.4012112	0.13492098	-2.9736755	0.01527879	0.66950864
M - ZnCu	Faecalibacterium	-0.7493485	0.26744944	-2.8018323	0.02602334	0.47267442
M - ZnCu	Roseburia	-0.7794952	0.25026243	-3.1147113	0.01029925	0.45863747
M - ZnCu	Treponema	2.79662947	0.74336141	3.76213968	0.00107451	16.389313
M - ZnCu	Oscillibacter	-1.4855684	0.3382575	-4.3918271	7.11E-05	0.22637363
M - ZnCu	Porphyromonadaceae_unclassified	-1.1365224	0.33263108	-3.4167654	0.00337963	0.32093317
M - ZnCu	Olsenella	1.98922401	0.56687383	3.50911246	0.00252894	7.30985915
M - ZnCu	Butyricicoccus	0.76490792	0.25902375	2.95304164	0.0165773	2.1487965
M - ZnCu	Ruminococcus	-0.5667333	0.16511636	-3.4323265	0.00327959	0.56737589
M - ZnCu	Bifidobacterium	2.2042704	0.79761532	2.7635758	0.02915979	9.06363636
M - ZnCu	Selenomonas	2.66563313	0.59947487	4.4466136	7.13E-05	14.3770492
M - ZnCu	Clostridia_unclassified	1.9029851	0.43098873	4.41539411	0.00021892	6.70588235
M - ZnCu	Campylobacter	2.45958884	0.60834529	4.04308029	0.00039292	11.7
M - ZnCu	Desulfovibrio	2.60268969	0.86865068	2.99624435	0.01392845	13.5
M - ZnCu	Clostridium_XlVb	-1.540445	0.50212119	-3.067875	0.01084537	0.21428571

M - ZnCu	Clostridium_XlVa	-1.6094379	0.60403501	-2.6644779	0.03826044	0.2
M - ZnCu	Anaerovibrio	-2.2787744	0.25465513	-8.9484725	0	0.10240964
M - ZnCu	Parabacteroides	-1.3581235	0.47611974	-2.8524831	0.0219854	0.25714286
M - ZnCu	Gammaproteobacteria_unclassified	4.78749174	1.65951315	2.88487726	0.02014325	120
M - ZnCu	Faecalicoccus	-2.793208	0.67528503	-4.1363393	0.00015585	0.06122449
NTC - AB	Streptococcus	0.74675663	0.26842882	2.78195402	0.02801263	2.11014493
NTC - AB	Anaerovibrio	-1.9412912	0.19206673	-10.107379	0	0.14351852
NTC - ZnCu	Oscillibacter	-1.0977336	0.35265113	-3.1128031	0.00987795	0.33362637
NTC - ZnCu	Porphyromonadaceae_unclassified	-1.262393	0.34951026	-3.6118912	0.00155403	0.28297604
NTC - ZnCu	Olsenella	1.8412201	0.59382959	3.10058665	0.01049971	6.30422535
NTC - ZnCu	Bifidobacterium	2.59172065	0.83583121	3.10077036	0.01024605	13.3527273
NTC - ZnCu	Selenomonas	2.49469466	0.62772358	3.97419299	0.00037095	12.1180328
NTC - ZnCu	Clostridia_unclassified	1.82644503	0.44739085	4.08243713	0.00023809	6.21176471
NTC - ZnCu	Erysipelotrichaceae_unclassified	1.82866403	0.54885298	3.33179208	0.00498575	6.22556391
NTC - ZnCu	Campylobacter	2.35327821	0.63594609	3.70043661	0.00122548	10.52
NTC - ZnCu	Desulfovibrio	3.29583687	0.87263445	3.77688146	0.00076604	27
NTC - ZnCu	Anaerovibrio	-1.495679	0.1956628	-7.6441664	4.92E-14	0.22409639
NTC - ZnCu	Peptococcus	1.08856195	0.2769759	3.9301685	0.00054426	2.97
NTC - ZnCu	Faecalicoccus	-1.9177393	0.54732992	-3.5038086	0.00241794	0.14693878

a) Delta Estimates were calculated using glht function in the multcomp package in R. The glht function calculates the estimates by subtracting the estimate of X2 (second treatment group in the column “Comparison”) from the estimate of X1 (first treatment group in the column “Comparison”). Estimates for each treatment group were obtained with Negative binomial glm models, which were used as the input for the glht function. See the provided R-script (electronic supplemental material) for the procedure.

b) Fold Changes were calculated by taking the exponential function from the Delta Estimate (due to the logarithmic link function that was used in with negative binomial glm models). This was done in R using the command exp(Delta.Estimate). See the provided R-script (electronic supplemental material) for the procedure. Relative abundances of all genera are presented in Supplemental figure **S3B**

Table S4. Pairwise comparisons of gamma distribution GLMs of relative abundances of each ARG or MGE between treatment groups.

Comparison (X_1 - X_2)	Gene	Delta Estimate (X1 - X2) [a]	Std. Error	z-value	p.adjusted	Fold Change (X1 / X2) [b]
AB – ZnCu	tetM	-1.9609786	0.56562766	-3.4669071	0.00294252	0.14072065
AB - ZnCu	blaSFO	-1.6624443	0.6172798	-2.6931779	0.03566898	0.18967479
AB - ZnCu	tet(32)	-0.8667544	0.29147371	-2.9736966	0.01532269	0.42031351
AB - ZnCu	tetA	-3.3801931	0.80640588	-4.1916772	0.00012203	0.03404088
AB - ZnCu	aac(6)-Iy	-2.2241923	0.64701255	-3.4376339	0.00316533	0.10815474
AB - ZnCu	orf37-IS26	1.70692972	0.27849818	6.12905162	2.66E-09	5.51201204
AB - ZnCu	aac3ia	1.64899726	0.62668685	2.63129385	0.04215185	5.20176117
AB - ZnCu	aadA17	3.03054434	0.75440944	4.01710817	0.00036428	20.7085019
AB - ZnCu	aph(3)-ia	1.6305567	0.38277192	4.25986499	0.00014771	5.10671682
M - AB	erm(A)	-2.1113086	0.76595471	-2.7564406	0.0297697	0.12107941
M - AB	dfrA15	2.48130513	0.70164466	3.53641277	0.00223079	11.9568595
M - AB	ISEfm1-Entero	-2.9992904	0.98225854	-3.0534633	0.01213616	0.04982241
M - AB	mcr-1	4.2850184	0.79531544	5.38782244	2.35E-07	72.6038824
M - AB	IS1247	-2.8561563	0.93354463	-3.0594749	0.01153335	0.0574893
M - AB	tet(32)	0.9720145	0.3057002	3.17963315	0.00818034	2.64326396
M - AB	vanRB	5.93955828	0.87592439	6.78090298	2.52E-11	379.767142
M - AB	bacA	-1.8909589	0.62089051	-3.0455593	0.01254414	0.15092702
M - AB	cmr	-2.9181191	0.81540135	-3.5787519	0.00198127	0.05403523
M - AB	IS1111	1.07880591	0.4100313	2.63103307	0.04210773	2.94116543
M - AB	tetW	0.59294255	0.11487403	5.16167619	1.79E-06	1.80930456
M - AB	cmlA1	2.9568897	0.5889674	5.02046413	2.50E-06	19.2380425
M - AB	ermT	2.28000567	0.54303264	4.19865312	0.00013153	9.77673588
M - AB	tetA	4.40984486	0.84576562	5.2140271	1.32E-06	82.2567008
M - AB	orf37-IS26	-1.7069297	0.29209135	-5.8438214	2.38E-08	0.18142196
M - AB	cmlA5	3.00497288	0.59048343	5.08900462	2.59E-06	20.1856687
M - AB	QnrB4	1.78597148	0.32353984	5.52009763	1.68E-07	5.96537237
M - AB	mphA	-2.1385063	0.53931174	-3.9652507	0.00035192	0.11783072
M - AB	aac(3)-Xa	3.47371474	0.36875121	9.42021236	0	32.256344
M - AB	ant6-ia	-0.8613593	0.21763422	-3.9578302	0.00049996	0.42258728
M - AB	Aac6-Aph2	1.5327412	0.30740967	4.98598883	4.25E-06	4.63085353
M - AB	aph(3)-ia	-1.6305567	0.40145458	-4.0616219	0.00025999	0.19582053
M - AB	tetC	-1.9095796	0.69594238	-2.743876	0.03098174	0.14814265
M - AB	acc3-iva	-2.5851379	0.72497665	-3.5658223	0.00199241	0.07538568
M - NTC	dfra21	-3.9631864	0.7464272	-5.3095418	4.30E-07	0.01900247
M - NTC	mcr-1	3.11136771	0.79531544	3.9121178	0.00054159	22.4517308
M - NTC	IncF	-2.7468676	0.71209149	-3.8574644	0.00068427	0.06412843
M - NTC	IS613	-0.5945431	0.19546268	-3.0417219	0.01234019	0.55181463

M - NTC	int1-a-marko	-4.0443799	0.74755476	-5.4101454	3.42E-07	0.01752057
M - NTC	vanYB	-1.6261959	0.40225007	-4.0427485	0.00037319	0.19667634
M - NTC	IS1247	-2.5124873	0.93354463	-2.6913414	0.03556148	0.08106635
M - NTC	blaSFO	-2.8016459	0.64740851	-4.3274777	0.00012122	0.06071006
M - NTC	vanHB	-1.8205826	0.46912614	-3.8807956	0.00056227	0.16193138
M - NTC	vanRB	4.19033987	0.87592439	4.78390594	1.58E-05	66.0452339
M - NTC	IS200	-2.1107617	0.78217107	-2.6985934	0.03530454	0.12114566
M - NTC	sugE	-2.2099317	0.67741053	-3.2623227	0.00604713	0.10970814
M - NTC	IS1111	1.07880591	0.4100313	2.63103307	0.04215758	2.94116543
M - NTC	dfrA12	-3.5203626	0.73837227	-4.7677341	9.18E-06	0.0295887
M - NTC	tetW	0.31333733	0.11487403	2.72766025	0.03253207	1.36798291
M - NTC	cmlA1	2.9568897	0.5889674	5.02046413	2.29E-06	19.2380425
M - NTC	aac3ia	1.89166903	0.65727471	2.8780493	0.02077131	6.63042586
M - NTC	cmlA5	3.00497288	0.59048343	5.08900462	3.01E-06	20.1856687
M - NTC	QnrB4	1.78597148	0.32353984	5.52009763	9.52E-08	5.96537237
M - NTC	mphA	-1.4728425	0.53931174	-2.7309668	0.03232183	0.22927286
M - NTC	fabK	5.66404178	1.09149277	5.18926182	1.53E-06	288.311582
M - NTC	aac(3)-Xa	3.47371474	0.36875121	9.42021236	0	32.256344
M - NTC	ant6-ia	-0.7219647	0.21763422	-3.3173308	0.0050286	0.48579688
M - NTC	Aac6-Aph2	1.5327412	0.30740967	4.98598883	3.46E-06	4.63085353
M - NTC	aadA17	4.68465257	0.7912313	5.92071191	9.04E-09	108.272649
M - NTC	strA	3.30889729	0.98127942	3.37202353	0.00437277	27.3549443
M - NTC	spcN	-1.9089525	0.49374891	-3.8662415	0.0006272	0.14823558
M - NTC	acc3-iva	-2.1937907	0.72497665	-3.0260156	0.01318488	0.11149331
M - ZnCu	dfrA15	2.48130513	0.70164466	3.53641277	0.00217154	11.9568595
M - ZnCu	ISEfm1-Entero	-3.2048101	0.98225854	-3.262695	0.00593547	0.04056661
M - ZnCu	mdth	2.9246563	0.86237088	3.39141356	0.00380529	18.6278225
M - ZnCu	erm(B)	0.93109457	0.33870954	2.74894695	0.03070325	2.53728489
M - ZnCu	mcr-1	4.2850184	0.79531544	5.38782244	3.78E-07	72.6038824
M - ZnCu	IS613	-0.5225922	0.19546268	-2.6736165	0.03762232	0.5929814
M - ZnCu	vat(E)	-1.2091678	0.37578186	-3.2177387	0.00677412	0.29844553
M - ZnCu	tetM	-1.743174	0.59323529	-2.9384193	0.01727917	0.17496418
M - ZnCu	blaSFO	-1.6624443	0.64740851	-2.5678444	0.04998973	0.18967479
M - ZnCu	tetU	2.29801572	0.69439174	3.30939381	0.00522197	9.95441055
M - ZnCu	vanRB	5.93955828	0.87592439	6.78090298	4.37E-11	379.767142
M - ZnCu	bacA	-2.1036734	0.62089051	-3.3881552	0.00401755	0.12200742
M - ZnCu	cmr	-2.1813192	0.81540135	-2.6751478	0.03746989	0.11289251
M - ZnCu	IS1111	1.07880591	0.4100313	2.63103307	0.04184856	2.94116543
M - ZnCu	tetW	0.39580496	0.11487403	3.44555648	0.00324454	1.48557954

M - ZnCu	cmlA1	2.9568897	0.5889674	5.02046413	3.46E-06	19.2380425
M - ZnCu	ermT	2.12523835	0.54303264	3.91364754	0.000564	8.37489337
M - ZnCu	aac(6)-Iy	-2.2241923	0.67859249	-3.2776553	0.00590438	0.10815474
M - ZnCu	aac3ia	1.89166903	0.65727471	2.8780493	0.02082748	6.63042586
M - ZnCu	cmlA5	3.00497288	0.59048343	5.08900462	3.37E-06	20.1856687
M - ZnCu	QnrB4	1.78597148	0.32353984	5.52009763	1.33E-07	5.96537237
M - ZnCu	mphA	-2.0535919	0.53931174	-3.8078013	0.00084003	0.12827332
M - ZnCu	aac(3)-Xa	3.47371474	0.36875121	9.42021236	0	32.256344
M - ZnCu	ant6-ia	-0.8695084	0.21763422	-3.9952742	0.00036814	0.41915756
M - ZnCu	Aac6-Aph2	1.5327412	0.30740967	4.98598883	4.42E-06	4.63085353
M - ZnCu	aadA17	4.68465257	0.7912313	5.92071191	8.40E-09	108.272649
M - ZnCu	tetC	-1.958219	0.69594238	-2.8137659	0.02511551	0.14110952
NTC - AB	ere(A)	-3.7985065	1.118659	-3.3955892	0.00364477	0.02240421
NTC - AB	dfra21	3.96318637	0.71169041	5.56869437	5.08E-07	52.6247409
NTC - AB	erm(A)	-3.4796663	0.73030916	-4.7646483	1.12E-05	0.03081769
NTC - AB	IncF	2.74686756	0.6789526	4.04574274	0.00033225	15.5937089
NTC - AB	int1-a-marko	4.04437991	0.7127655	5.67420832	5.89E-08	57.0757829
NTC - AB	vanYB	1.62619587	0.3835304	4.24007036	0.00011458	5.0844958
NTC - AB	vat(E)	-0.9633283	0.35829394	-2.6886537	0.03557084	0.38162061
NTC - AB	blaSFO	2.80164588	0.6172798	4.53869688	3.12E-05	16.471735
NTC - AB	vanHB	1.82058264	0.44729422	4.07021273	0.0002616	6.17545545
NTC - AB	VanB	1.4812986	0.56054483	2.64260507	0.04109909	4.39865408
NTC - AB	bacA	-1.8909589	0.59199588	-3.1942096	0.00729479	0.15092702
NTC - AB	sugE	2.20993173	0.6458856	3.42155287	0.00316531	9.11509408
NTC - AB	cmr	-2.9181191	0.77745468	-3.7534266	0.00096566	0.05403523
NTC - AB	dfrA12	3.52036262	0.70401033	5.0004417	2.61E-06	33.7966817
NTC - AB	ermT	1.43379945	0.5177613	2.76922867	0.02844954	4.19460614
NTC - AB	tetA	2.23750725	0.80640588	2.77466635	0.02830619	9.36994519
NTC - AB	orf37-IS26	-1.7069297	0.27849818	-6.1290516	2.37E-09	0.18142196
NTC - AB	aac3ia	-1.6489973	0.62668685	-2.6312938	0.04216712	0.19224258
NTC - AB	aadD	-2.5570934	0.82041991	-3.1168105	0.01000558	0.07752976
NTC - AB	fabK	-6.4623226	1.04069753	-6.209607	1.87E-08	0.00156117
NTC - AB	aadA17	-3.0305443	0.75440944	-4.0171082	0.00035953	0.04828935
NTC - AB	aph(3)-ia	-1.6305567	0.38277192	-4.259865	0.00014469	0.19582053
NTC - AB	spcN	1.9089525	0.47077111	4.05494826	0.00026519	6.74601867
NTC - ZnCu	dfra21	3.96318637	0.71169041	5.56869437	1.47E-07	52.6247409
NTC - ZnCu	erm(A)	-2.2898479	0.73030916	-3.1354501	0.00921967	0.10128186
NTC - ZnCu	mdth	3.19193363	0.82223837	3.8820052	0.00065477	24.3354377
NTC - ZnCu	erm(B)	0.84582312	0.32294688	2.61907817	0.04367295	2.32989481

NTC - ZnCu	IncF	2.74686756	0.6789526	4.04574274	0.00030468	15.5937089
NTC - ZnCu	int1-a-marko	4.04437991	0.7127655	5.67420832	1.30E-07	57.0757829
NTC - ZnCu	vanYB	1.62619587	0.3835304	4.24007036	0.00011774	5.0844958
NTC - ZnCu	vat(E)	-1.2481406	0.35829394	-3.4835659	0.00276248	0.28703803
NTC - ZnCu	vanHB	1.82058264	0.44729422	4.07021273	0.00022799	6.17545545
NTC - ZnCu	VanB	1.4812986	0.56054483	2.64260507	0.04103194	4.39865408
NTC - ZnCu	tetU	2.15358493	0.66207655	3.25277333	0.00610526	8.61568974
NTC - ZnCu	IS200	2.11076166	0.74577086	2.83030859	0.02401747	8.25452605
NTC - ZnCu	bacA	-2.1036734	0.59199588	-3.5535271	0.00222767	0.12200742
NTC - ZnCu	sugE	2.20993173	0.6458856	3.42155287	0.00365646	9.11509408
NTC - ZnCu	cmr	-2.1813192	0.77745468	-2.8057187	0.02591365	0.11289251
NTC - ZnCu	dfrA12	3.52036262	0.70401033	5.0004417	3.54E-06	33.7966817
NTC - ZnCu	aadA2	1.89169834	0.72517442	2.60861151	0.04472002	6.6306202
NTC - ZnCu	aac(6)-Iy	-2.2241923	0.64701255	-3.4376339	0.00315928	0.10815474
NTC - ZnCu	aadD	-2.6220232	0.82041991	-3.1959527	0.00703458	0.07265572
NTC - ZnCu	fabK	-5.6767979	1.04069753	-5.454801	2.60E-07	0.00342451
NTC - ZnCu	spcN	1.9089525	0.47077111	4.05494826	0.00026009	6.74601867
NTC - ZnCu	aph(2)-Id	-0.4948545	0.19239134	-2.5721245	0.04936651	0.60965962

a) Delta Estimates were calculated using glht function in the multcomp package in R. The glht function calculates the estimates by subtracting the estimate of X2 (second treatment group in the column “Comparison”) from the estimate of X1 (first treatment group in the column “Comparison”). Estimates for each treatment group were obtained with Gamma glm models, which were used as the input for the glht function. See the provided R-script (electronic supplemental material) for the procedure.

b) Fold Changes were calculated by taking the exponential function from the Delta Estimate (due to the logarithmic link function that was used in with Gamma glm models). This was done in R using the command exp(Delta.Estimate). See the provided R-script (electronic supplemental material) for the procedure. Relative abundances of all genera are presented in Supplemental figure **S3C**

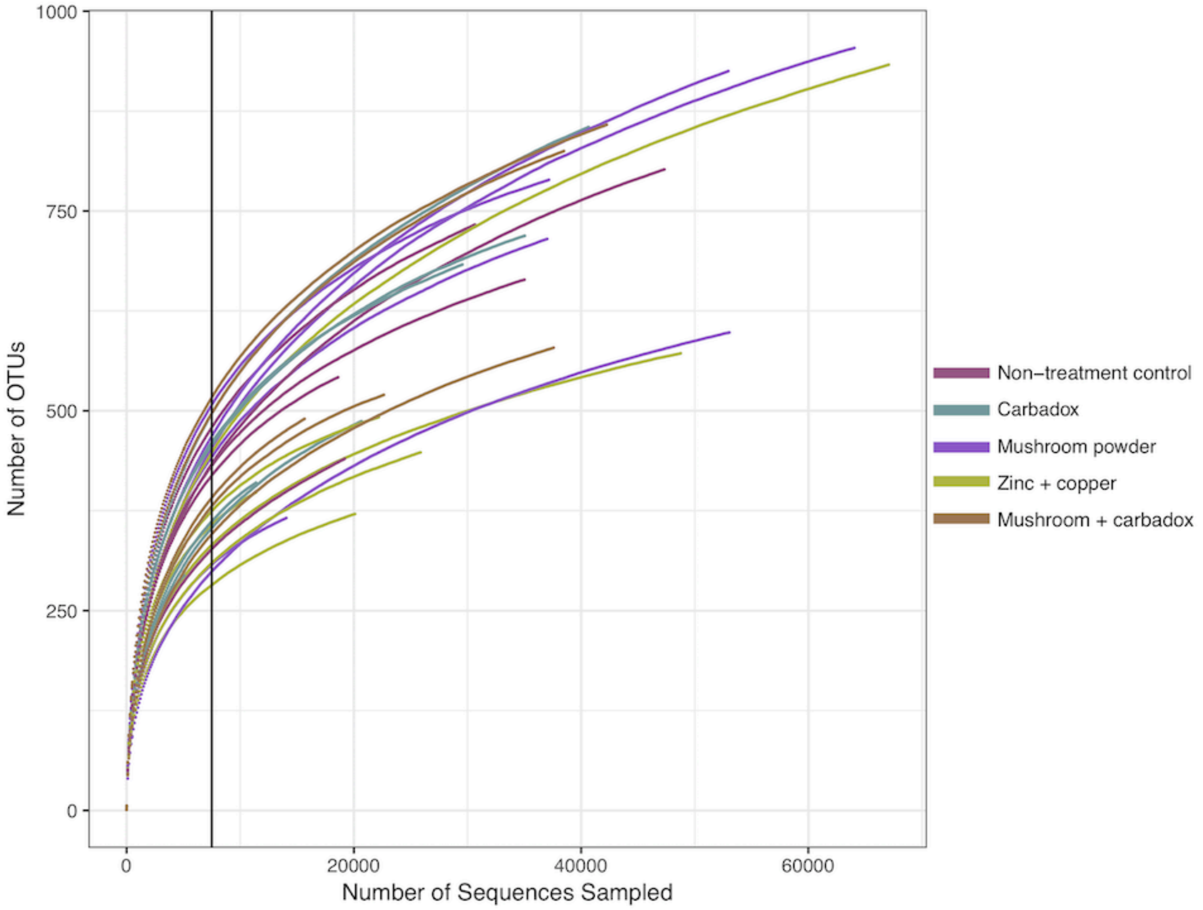

Fig. S4. Rarefaction curves. OTU collection curves determined from sequence analysis. Each line represents one sample. Vertical line shows the subsampling cutoff: 7500 sequences

Table S5. Assays that had unspecific amplification. Ct values in the negative control and mean Ct-values in samples.

Assay	Ct in the negative control	Mean Ct in samples
16S old 1_1	24.14393	11.10064
blaOXY-1_1118	23.06278	22.71912
cmlV_911	26.30517	25.85088
czcA_1536	16.75728	17.97052
fabK_1520	25.9036	19.75017
intI1F165_clinical_359	25.72579	23.82255
tetPA_1507	24.76249	NA
